# JT002, a small molecule inhibitor of the NLRP3 inflammasome for the treatment of autoinflammatory disorders

**DOI:** 10.1038/s41598-023-39805-z

**Published:** 2023-08-19

**Authors:** Geza Ambrus-Aikelin, Katsuyuki Takeda, Anthony Joetham, Milos Lazic, Davide Povero, Angelina M. Santini, Rama Pranadinata, Casey D. Johnson, Matthew D. McGeough, Federico C. Beasley, Ryan Stansfield, Christopher McBride, Lynnie Trzoss, Hal M. Hoffman, Ariel E. Feldstein, Jeffrey A. Stafford, James M. Veal, Gretchen Bain, Erwin W. Gelfand

**Affiliations:** 1 Jecure Therapeutics, San Diego, CA USA; 2 Department of Pediatrics, National Jewish Health, Denver, CO USA; 3 Division of Gastroenterology and Hepatology, Mayo Clinic, 200 First Street SW, Rochester, MN USA; 4 Department of Pediatrics, University of California San Diego, La Jolla, CA USA

**Keywords:** Drug discovery, Diseases

## Abstract

The NLRP3 inflammasome is an intracellular, multiprotein complex that promotes the auto-catalytic activation of caspase-1 and the subsequent maturation and secretion of the pro-inflammatory cytokines, IL-1β and IL-18. Persistent activation of the NLRP3 inflammasome has been implicated in the pathophysiology of a number of inflammatory and autoimmune diseases, including neuroinflammation, cardiovascular disease, non-alcoholic steatohepatitis, lupus nephritis and severe asthma. Here we describe the preclinical profile of JT002, a novel small molecule inhibitor of the NLRP3 inflammasome. JT002 potently reduced NLRP3-dependent proinflammatory cytokine production across a number of cellular assays and prevented pyroptosis, an inflammatory form of cell death triggered by active caspase-1. JT002 demonstrated in vivo target engagement at therapeutically relevant concentrations when orally dosed in mice and prevented body weight loss and improved inflammatory and fibrotic endpoints in a model of Muckle–Wells syndrome (MWS). In two distinct models of neutrophilic airway inflammation, JT002 treatment significantly reduced airway hyperresponsiveness and airway neutrophilia. These results provide a rationale for the therapeutic targeting of the NLRP3 inflammasome in severe asthma and point to the use of JT002 in a variety of inflammatory disorders.

## Introduction

Key mediators of innate immunity include the NOD-like family of receptors (NLRs) that serve as sensors of pathogen-associated molecular patterns (PAMPs) and damage- (or danger-) associated molecular patterns (DAMPs)^1^. Upon activation, NLRs self-assemble into caspase-1 activating macromolecular structures called inflammasomes that control the maturation of interleukin-1 beta (IL-1β) and interleukin-18 (IL-18)^1–3^. Active caspase-1 also cleaves the intracellular protein Gasdermin D (GSDMD) and releases an N-terminal fragment that oligomerizes and forms pores in the plasma membrane to induce a form of inflammatory cell death termed pyroptosis^4^. Loss of membrane integrity facilitates the release of IL-1β and IL-18 and other soluble mediators, such as IL-1α, from the cell^5^.

The NLRP3 sensor, one of the most well-characterized NLR family members, is activated by a range of stimuli including sterile inflammatory signals. The canonical pathway of NLRP3 inflammasome activation occurs in a two-step process that is initiated by a priming step through an NF-κB-activating signal, such as Toll-like receptor 4 (TLR4) stimulation. This signal induces transcription of the NLRP3, pro-IL-1β and pro-IL-18 genes and promotes post-translational modifications of NLRP3 required for optimal activation^2^. The primed NLRP3 inflammasome is subsequently activated by molecules or substances that include ATP, pore-forming toxins, crystalline/particulate material, and nucleic acids that induce the formation of NLRP3 oligomers. The NLRP3 oligomer scaffold then organizes the normally diffuse adapter protein, apoptosis associated speck-like protein containing a CARD (ASC), into dense ASC specks that can be visualized by imaging. Subsequently, procaspase-1 is recruited to the inflammasome and autocatalytic cleavage leads to the formation of active caspase-1^6^.

Two additional and distinct pathways of NLRP3 activation, designated the non-canonical and alternative pathways, also lead to IL-1β and IL-18 maturation. The non-canonical pathway involves the recognition of intracellular LPS by caspase-11 in mice or caspase-4, -5 in humans that results in the cleavage of the pyroptotic substrate, Gasdermin D^7^. NLRP3 is then activated downstream of caspase-11/-4/-5 activation as a result of membrane disruption by the GSDMD pores and efflux of potassium ions, thus leading to the NLRP3-mediated activation of caspase-1 and the maturation and release of IL-1β and IL-18^8,9^. The alternative pathway of NLRP3 activation has been demonstrated in human monocytes, but not macrophages or rodent monocytes. Activation of the alternative NLRP3 pathway results in the release of mature IL-1β in response to TLR4 ligands alone and in the absence of a secondary stimulus^10^. Although mature IL-1β is released, activation of the alternative NLRP3 pathway occurs in the absence of pyroptosis and ASC speck formation and is independent of potassium efflux^8^.

The contribution of NLRP3 activation to human disease is highlighted by a family of inherited, autosomal dominant, autoinflammatory diseases referred to as cryopyrin-associated periodic syndromes (CAPS)^11^. Gain-of-function point mutations that confer constitutive activation of NLRP3 have been identified as the cause of the three syndromes that comprise the CAPS family: familial cold autoinflammatory syndrome (FCAS), Muckle-Wells syndrome (MWS), and neonatal onset multisystem inflammatory disease (NOMID), which have overlapping symptoms of fever, rash and arthralgia but differ in terms of severity, organ manifestations and environmental triggers^12–14^. As a key sensor of sterile inflammatory signals, NLRP3 has been implicated in the pathophysiology of multiple other autoimmune and inflammatory diseases that are initiated or maintained due to the inappropriate presence of these sterile signals^15^. NLRP3 has been linked to the pathophysiology of rheumatoid arthritis (RA)^16,17^, systemic lupus erythematosus (SLE)^18–20^, gout^17,21^, nonalcoholic steatohepatitis (NASH)^22–25^, Alzheimer’s disease (AD)^17,26,27^, multiple sclerosis (MS)^28–33^, atherosclerosis^17,34,35^ and asthma and airway inflammation^36–38^. The involvement of NLRP3 in mediating a variety of inflammatory-related diseases is supported by preclinical data generated with MCC950, a small molecule inhibitor of NLRP3, which has shown efficacy in experimental models, including multiple sclerosis^29^, Alzheimer’s disease^26^, lupus nephritis^19^, rheumatoid arthritis^39^, MWS^29^, and colitis^40^. As such, there is considerable clinical interest in NLRP3 inhibitors for treating autoimmune and inflammatory-related diseases.

Asthma is a chronic, heterogeneous lung disorder characterized by persistent inflammation of the airways leading to airway hyperresponsiveness and remodeling. The majority of asthma patients can effectively control their symptoms with steroid therapies, however, some patients with severe disease fail to achieve adequate symptom control despite high doses of inhaled and systemic corticosteroids. NLRP3 inflammasome activation and exaggerated IL-1β production have been implicated in the pathogenesis of severe, steroid-resistant neutrophilic asthma. Sputum from neutrophilic asthmatics has been shown to exhibit elevated gene expression of NLRP3, caspase-1 and IL-1β and increased IL-1β protein concentration as compared to samples from eosinophilic asthmatics, patients with mild asthma or healthy controls^37,41,42^. Additionally, sputum expression of NLRP3 and IL-1β mRNA correlated with increased neutrophil numbers, severity of airflow obstruction and reduced disease control in these asthma patients. The roles and therapeutic targeting of the NLRP3 inflammasome, caspase-1, and IL-1β have been investigated in animal models of steroid-resistant, neutrophilic asthma, including two murine infection-induced models of severe, steroid-resistant asthma^37^. Treatment with inhibitors of IL-1β, caspase-1, and NLRP3 directly suppressed airway inflammation and airway hyperresponsiveness in these models and NLRP3 inhibition with intraperitoneal (i.p.) administration of MCC950 exerted more potent effects as compared with inhibiting caspase-1 or IL-1β^37^. In a murine house dust mite model of severe corticosteroid-resistant asthma, i.p. administration of MCC950 completely prevented the development of airway hyperresponsiveness and reduced bronchoalveolar lavage neutrophilia and IL-1β concentrations^42^.

Here we characterize the orally bioavailable small molecule JT002, and demonstrate that it is a potent and selective inhibitor of NLRP3 inflammasome assembly. JT002 has superior potency in cellular and whole blood assays as compared to MCC950 and displays good in vivo pharmacokinetic and pharmacodynamic properties. We demonstrate in vivo efficacy of JT002 after oral dosing in a mouse model of CAPS, as well as in two models of neutrophilic airway inflammation.

## Results

### JT002 is a potent and selective inhibitor of NLRP3 mediated cytokine production and pyroptosis

**Figure 1:**
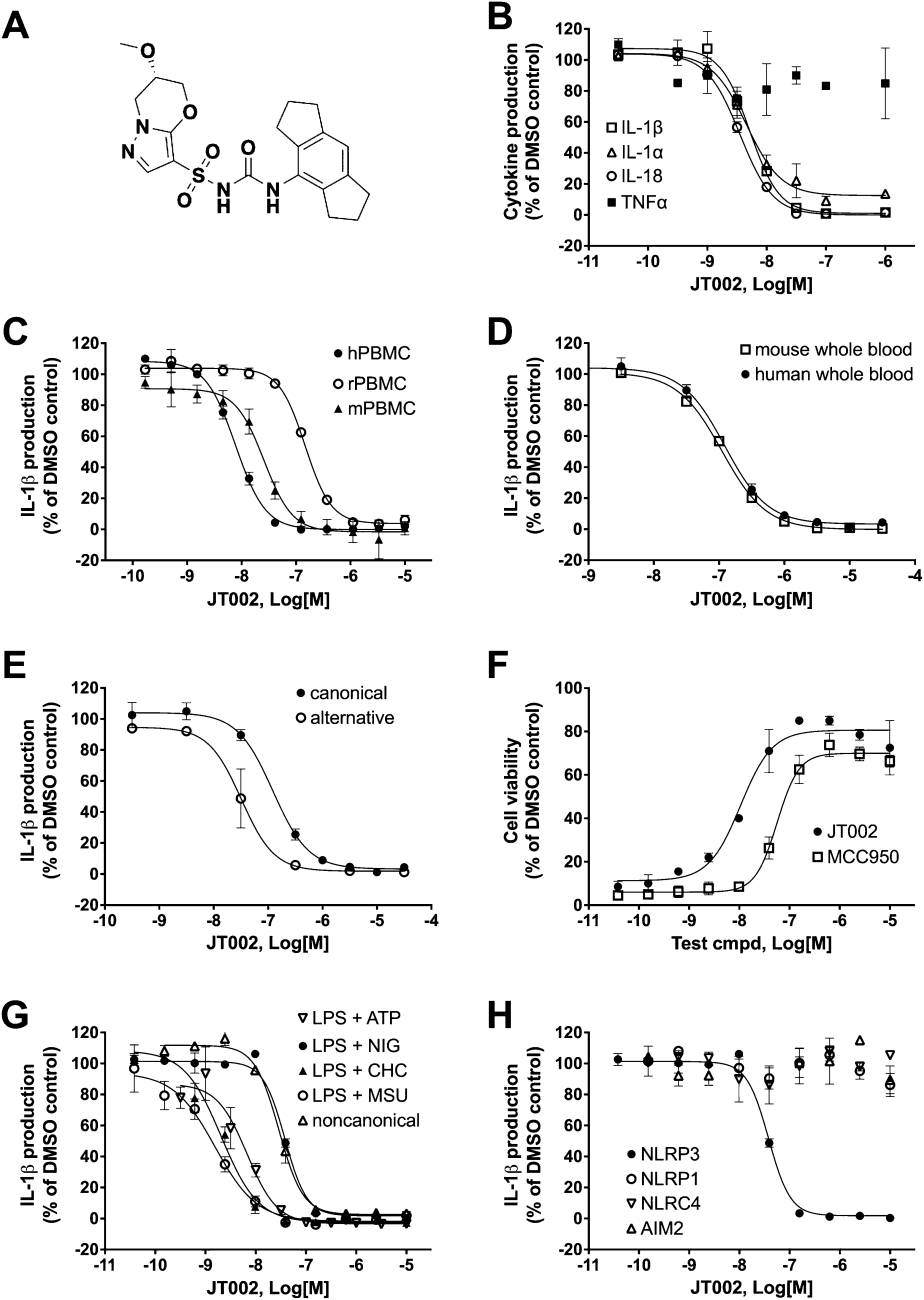
Cellular potency and selectivity of JT002. (**A**) Structure of JT002. (**B**) Inhibition of cytokine production by JT002 in human PBMCs stimulated by LPS + ATP. (**C**) Inhibition of IL-1β production by JT002 in PBMCs from human, rat and mouse stimulated by LPS + nigericin. (**D**) Inhibition of IL-1β production by JT002 in LPS + ATP stimulated human and mouse whole blood. (**E**) Inhibition of IL-1β production by JT002 in LPS + ATP stimulated (canonical) and LPS stimulated (alternative) human whole blood. (**F**) Inhibition of pyroptotic cell death by JT002 as measured by cellular ATP content in LPS + nigericin stimulated mouse BMDMs. (**G**) Inhibition of IL-1β production by JT002 in mouse BMDMs stimulated by a variety of NLRP3 activators. (**H**) Inhibition of IL-1β production by JT002 in mouse BMDMs using conditions that activate the NLRP1, NLRP3, NLRC4 and AIM2 inflammasomes. (**B–H**) Graphs show mean and standard error of the mean of 2–4 independent experiments.

The potency and selectivity of JT002 (Fig. 1A) in inhibiting NLRP3-dependent processes was tested in a number of primary cell-based assays using lipopolysaccharide (LPS) as a priming signal and a variety of secondary activators, including adenosine triphosphate (ATP), cholesterol crystals (CHC), monosodium urate (MSU) crystals and the pore-forming toxin, nigericin. In human peripheral blood mononuclear cells (hPBMCs) treated with LPS and activated with ATP, JT002 showed concentration-dependent inhibition of interleukin-1α (IL-1α), interleukin-1β (IL-1β) and interleukin-18 (IL-18) production with mean IC_50_ values of 3 nM, 4 nM and 4 nM, respectively, with no effect on tumor necrosis factor alpha (TNFα) production at JT002 concentrations up to 1 µM (Table 1, Fig. 1B). The potency of JT002 in blocking NLRP3-mediated IL-1β production was also tested in PBMCs from multiple species for a cross-species comparison. Under LPS plus nigericin stimulating conditions, JT002 inhibited IL-1β production from human, mouse and rat PBMCs with mean IC_50_ values of 6, 25 and 159 nM, respectively. Similar IC_50_ values were observed when either ATP or nigericin was used as the secondary stimulus (Table 1, Fig. 1C). Potency of JT002 in whole blood was evaluated as a more predictive measure of its in vivo potency. In human and mouse blood stimulated with LPS plus ATP, JT002 showed concentration-dependent inhibition of IL-1β production with mean IC_50_ values of 100 nM and 94 nM, respectively (Table 1, Fig. 1D). We also investigated the effect of JT002 on inhibiting the alternative pathway of NLRP3 activation using only LPS stimulation of human whole blood. JT002 potently inhibited IL-1β production under the alternative NLRP3 activating conditions with a mean IC_50_ value of 32 nM (Table 1, Fig. 1E). This IC_50_ value is approximately 3-lower than the IC_50_ value observed in human whole blood using canonical NLRP3 pathway activation (LPS + ATP).

**Table 1:** IC_50_ values for JT002 in a range of cell-based assays measuring multiple endpoints.

Species	Cell type	Stimulus	Readout	n	Geo Av IC_50_ (nM)	Geo SEM IC_50_ (x/ ÷)
Human	PBMC	LPS + ATP	IL-1β	4	4	1.3
			IL-18	3	4	1.2
			IL-1α	3	3	1.3
			TNFα	3	> 1000	N/A
		LPS + nigericin	IL-1β	17	6	1.1
			Caspase-1	1	8	N/A
	Whole blood	LPS + ATP	IL-1β	5	100	1.7
		LPS	IL-1β	2	32	2.0
Rat	PBMC	LPS + nigericin	IL-1β	2	159	1.0
Mouse	PBMC	LPS + ATP	IL-1β	3	7	1.0
		LPS + nigericin	IL-1β	2	25	1.0
	Whole blood	LPS + ATP	IL-1β	4	94	1.2
	BMDM	LPS + nigericin	IL-1β	3	23	1.7
			TNFα	3	> 10,000	N/A
			Viability	3	7	1.5
			ASC specks	1	3	N/A
		LPS + ATP	IL-1β	2	5	1.0
		LPS + cholesterol	IL-1β	2	2	1.3
		LPS + monosodium urate	IL-1β	2	3	2.8
		Pam3CSK4 + LPS	IL-1β	3	30	1.9

The number of independent experiments run on different days is displayed (n). Geometric average of IC_50_ values are shown in nanomolar concentration (Geo Av IC_50_), as well as, corresponding geometric standard error of the mean factors (Geo SEM IC_50_).

Activation of the canonical NLRP3 pathway triggers a form of cell death termed pyroptosis^43^. JT002 inhibited pyroptosis in LPS plus nigericin stimulated mouse bone-marrow derived macrophages (mBMDMs) as measured by cellular ATP content, with a mean IC_50_ value of 7 nM (Table 1, Fig. 1F). JT002 was approximately 4–fivefold more potent than MCC950 at inhibiting both pyroptosis of BMDMs and IL-1β production from human PBMCs (Fig. 1F, Supplementary Fig. 1A). We evaluated inhibition of NLRP3 downstream of distinct canonical NLRP3 triggers, including pathophysiological substances like cholesterol crystals (CHC) and monosodium urate (MSU) crystals. For these assays, potency was evaluated in LPS-primed mBMDMs by measuring IL-1β concentrations after stimulation with ATP, nigericin, CHC, or MSU crystals. For assessment of effects on the non-canonical NLRP3 pathway, mBMDMs were treated with Pam3CSK4 and transfected with LPS and IL-1 production in the culture supernatant measured^7^. JT002 blocked canonical NLRP3-dependent IL-1β production activated downstream of ATP, nigericin, CHC or MSU with mean IC_50_ values of 5, 23, 2 and 3 nM, respectively and inhibited non-canonical induced IL-1β production with a mean IC_50_ value of 30 nM (Table 1, Fig. 1G). Finally, we tested whether JT002 inhibits IL-1β production in mBMDMs upon selective activation of other NOD-like receptor inflammasomes and found no inhibition of IL-1β production after NLRP1, NLRC4 or AIM2 activation at concentrations up to 10 µM (Fig. 1H).

### JT002 blocks NLRP3 inflammasome complex formation and downstream pathway activation

**Figure 2:**
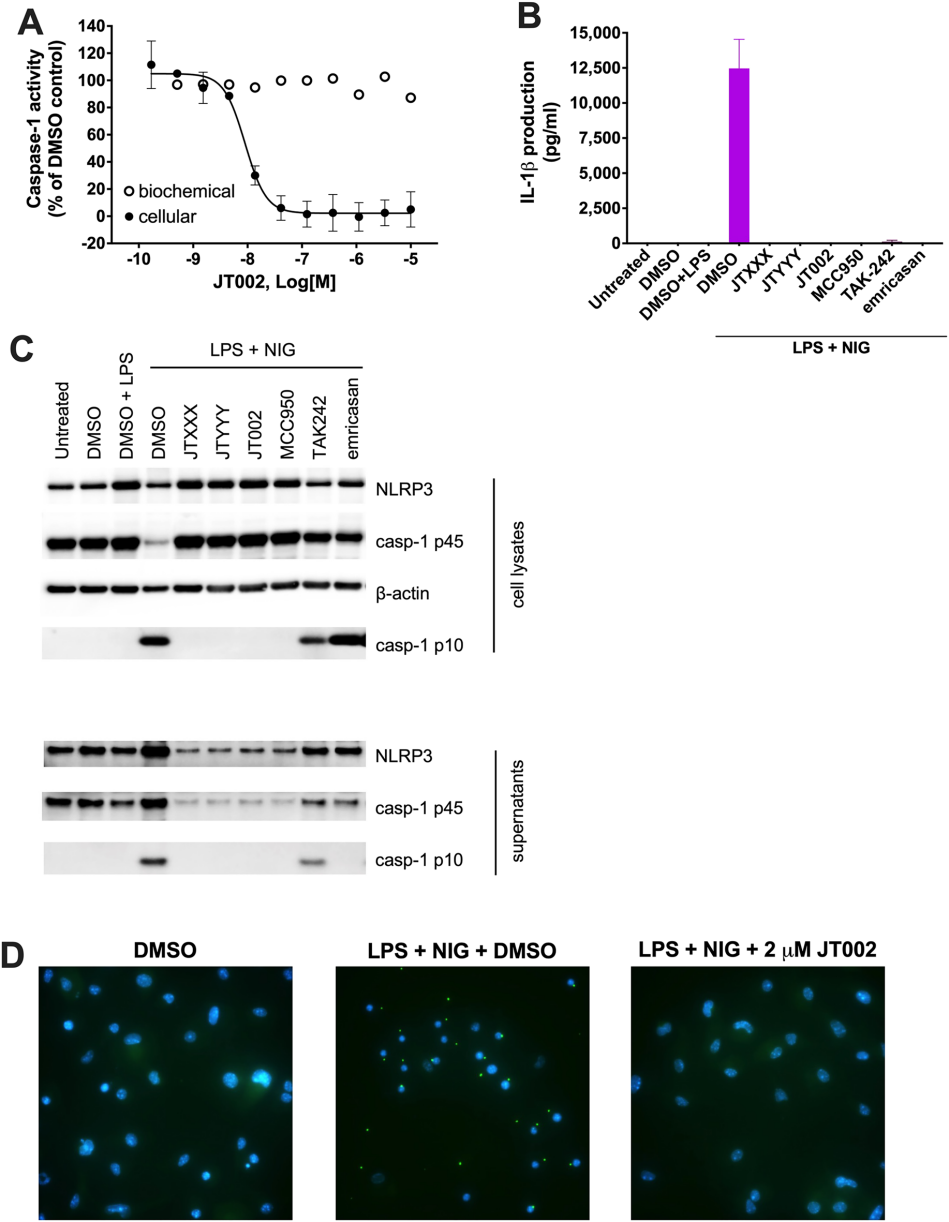
Cellular mechanism of action studies for JT002. (**A**) Inhibition of intracellular caspase-1 activity by JT002 in human PBMCs stimulated with LPS + nigericin. (**B**) Inhibition of IL-1β production by indicated compounds in the LPS + nigericin stimulated mouse BMDM samples used for Western blotting in panel (**C**). (**C**) Western blot analysis for NLRP3, procaspasae-1 (casp-1 p45), β-actin and active caspase-1 (casp-1 p10 in compound treated (10 µM) and LPS + nigericin stimulated mBMDMs lysates and corresponding supernatants. The blots reported are continuous, were run in the same gel and bands not relevant for the purpose of this figure were removed for better clarity and simplicity. Quantification of the actin normalized bands is shown in Supplementary Fig. 2 and complete (non-cropped) blots from different exposures are shown in Supplementary Figs. 6–11. (**D**) Immunofluorescence images of mouse BMDMs with indicated stimulus and compound treatment. Nuclei (blue) and ASC (green) are visualized. (**A,B**) Graphs show representative curves with mean and standard error of the mean plotted for any particular experiment.

Several mechanistic studies were performed to understand the precise mechanism of action of JT002. IL-1β and IL-18 maturation and release from cells is dependent on intracellular caspase-1 mediated enzymatic processing of the pro-interleukin forms, so we evaluated intracellular caspase-1 activity in human PBMCs activated by LPS plus nigericin^3^. Cellular caspase-1 activity was inhibited by JT002 with a mean IC_50_ value of 8 nM, which is in good correspondence to the IC_50_ value measured for the inhibition of IL-1β production under the same conditions (Table 1, Fig. 2A). However, JT002 is not a direct inhibitor of caspase-1 enzymatic activity as demonstrated in a cell-free biochemical assay (Fig. 2A). Upon assembly of the inflammasome, cellular caspase-1 is activated by autocatalytic cleavage of the p45 proenzyme into p10 and p33 subunits which form the active tetramer, with a second self-cleavage to produce p20 and p10 subunits^44^. Cellular caspase-1 activity is diminished if cleavage of pro-caspase-1 (p45) to the active caspase-1 subunits is prevented. In LPS plus nigericin treated mBMDMs, both JT002 and MCC950 at concentrations of 10 µM blocked formation of the p10 subunit and its release from the cell (Fig. 2C). By contrast, and consistent with their respective mechanisms of action, the caspase inhibitor emricasan^45^ and the TLR4 antagonist TAK242^46^ did not inhibit the autocatalytic cleavage of pro-caspase-1 into the p10 subunit (Fig. 2C). Loss of membrane integrity as a result of caspase-1 mediated pyroptosis is signified by the appearance of the p10 component in cell supernatants of the TAK242 treated samples, but not in the emricasan treated samples. These data are consistent with the ability of emricasan to inhibit caspase-1 enzymatic activity and block pyroptosis (Fig. 2B). Next, we examined whether JT002 blocks the assembly of the macromolecular NLRP3 inflammasome complex referred to as the ASC speck. Imaging studies were carried out after immunostaining for ASC in LPS plus nigericin treated mBMDMs in the presence or absence of JT002 (Fig. 2D). In unstimulated mBMDMs, ASC shows diffuse cytoplasmic staining, while LPS plus nigericin treatment promotes the formation of the ASC speck (Fig. 2D). Treatment with 2 µM JT002 prior to NLRP3 activation completely prevented ASC speck formation. Concentration–response experiments identified an IC_50_ of 3 nM for the blockade of NLRP3 inflammasome assembly which is in good agreement with the IC_50_ value for pyroptosis inhibition under the same experimental conditions (Table 1, Supplementary Fig. 1B).

### In vivo pharmacokinetic and pharmacodynamic profiles of JT002

The pharmacokinetics (PK) of JT002 were assessed in male Sprague–Dawley rats and male Beagle dogs after intravenous (i.v.) and oral (p.o.) administration (Table 2). After intravenous administration at 1 mg/kg, JT002 displayed low systemic clearance (Cl_p_) with values of 1.0 mL/min/kg and 1.8 mL/min/kg in rat and dog, respectively (Table 2). JT002 displayed a relatively short elimination half-life (t_1/2_) of 2.8 and 1.1 h in in rat and dog, respectively, and a low volume of distribution at steady state (V_ss_) with a value of 0.2 L/kg. The sodium salt form of JT002 was readily absorbed after oral administration, showing excellent bioavailability (73–100%), and reached a maximum plasma concentration of 15.6 µg/mL (36 µM) in rats at thirty minutes following a single 5 mg oral dose. In dogs, a maximum drug plasma concentration (C_max_) of 4.0 µg/mL (9 µM) was reached 1 h after a single 1 mg oral dose. Dose-adjusted exposures (dose-adjusted AUC) from oral administration were close to identical between rat and dog (11.8 versus 11.6) (Table 2).

**Table 2:** Pharmacokinetic parameters for JT002 in rat and dog after intravenous (i.v.) and oral (p.o.) administration.

	Rat		Dog	
	i.v	p.o	i.v	p.o
Dose (mg/kg)	1	5	1	1
T_max_ (h)	–	0.5*	–	1.0*
C_max_ (µg/mL)	10.6	15.6	7.0	4.0
t_1/2_ (h)	2.8	3.4	1.1	1.4
AUC (h·µg/mL)	16.2	59.2	10.6	11.6
Dose-adjusted AUC	16.2	11.8	10.6	11.6
Cl_pl_ (mL/min/kg)	1.0	–	1.8	–
V_ss_ (L/kg)	0.2	–	0.2	–
%F	–	73.2	–	100

*T*_*max*_ time of maximum concentration, *C*_*max*_ maximum concentration, *t*_*1/2*_ half-life, *AUC* area under the curve, *Cl*_*pl*_ plasma clearance, *V*_*ss*_ volume of distribution at steady state, *%F* oral bioavailability.

*Median.

**Figure 3:**
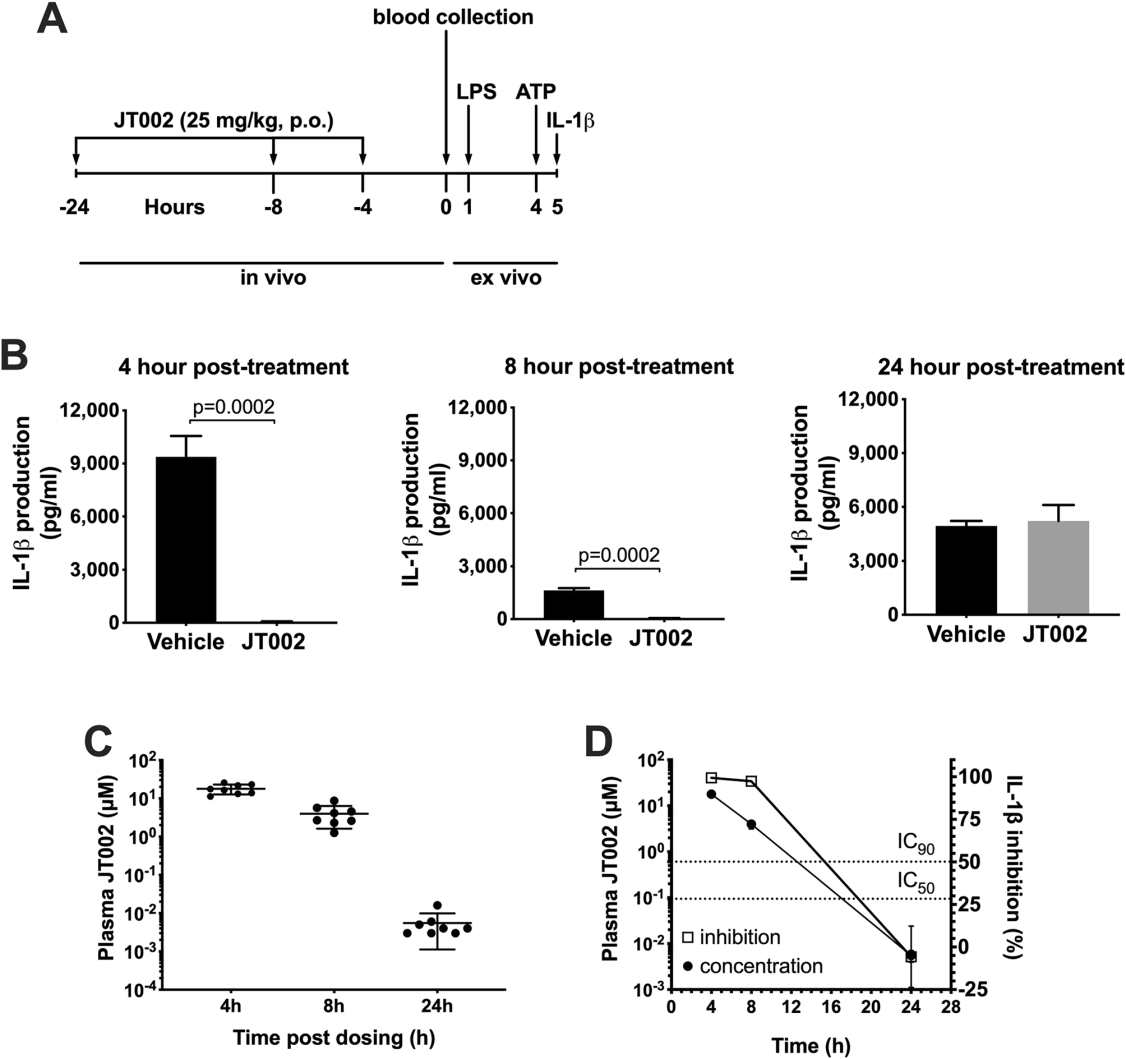
Pharmacokinetics and pharmacodynamic evaluation of JT002. (**A**) Study timeline of the pharmacodynamic assay. (**B**) Mean + SEM ex vivo stimulated plasma IL-1β concentrations from vehicle or 25 mg/kg JT002 treated mice collected at various timepoints post-dosing. (**C**) Mean $\pm$ SEM plasma JT002 concentrations at each time point post-dosing. (**D**) Correlation of mouse JT002 plasma concentrations and inhibition of ex vivo IL-1β production at each time point post dosing. Dotted lines indicate the in vitro mouse blood IC_50_ and IC_90_ values for JT002.

In vivo pharmacodynamics (PD) were evaluated in mice for determination of target engagement as this species is routinely used for assessment of preclinical efficacy. Treatment of fresh, whole blood with LPS plus ATP results in the NLRP3-dependent release of IL-1β, thus providing a robust method for tracking target engagement of NLRP3 inflammasome inhibitors in the circulation after oral dose administration^36,47^. In vivo target engagement using ex vivo stimulation of blood was evaluated in mice at various times after oral dosing of JT002. For each time point tested, one group of mice received a single oral dose of 25 mg/kg JT002 (n = 8/group) and another group received the vehicle control (n = 8/group). At the time of harvest, blood was collected from each animal and an aliquot removed and processed to plasma for determination of JT002 concentrations. The remaining blood was stimulated ex vivo with LPS plus ATP followed by analysis of IL-1β concentrations (Fig. 3A). As shown in Fig. 3B, LPS plus ATP treatment of fresh blood from vehicle dosed animals induced robust production of IL-1β with mean values ranging from 1629 to 9375 pg/mL. Oral dosing of 25 mg/kg JT002 resulted in $\ge$ 97% inhibition of ex vivo stimulated IL-1β production at 4- and 8-h post-dosing with no significant inhibition observed at 24-h post-dosing (Fig. 3B,D). Consistent with these findings, plasma concentrations of JT002 decreased over time, with mean concentrations of $\ge$ 4.0 µM through 8 h and a mean concentration of 6 nM at the 24-h sampling (Fig. 3C,D). The complete inhibition of IL-1β production at mean plasma concentrations of $\ge$ 4.0 µM and lack of inhibition at a mean plasma concentration of 6 nM is consistent with the in vitro mouse whole blood IC_50_ and IC_90_ values of 94 nM and 716 nM, respectively (Fig. 1D, Table 1).

### JT002 prevents disease progression in a mouse model of Muckle Wells syndrome

**Figure 4:**
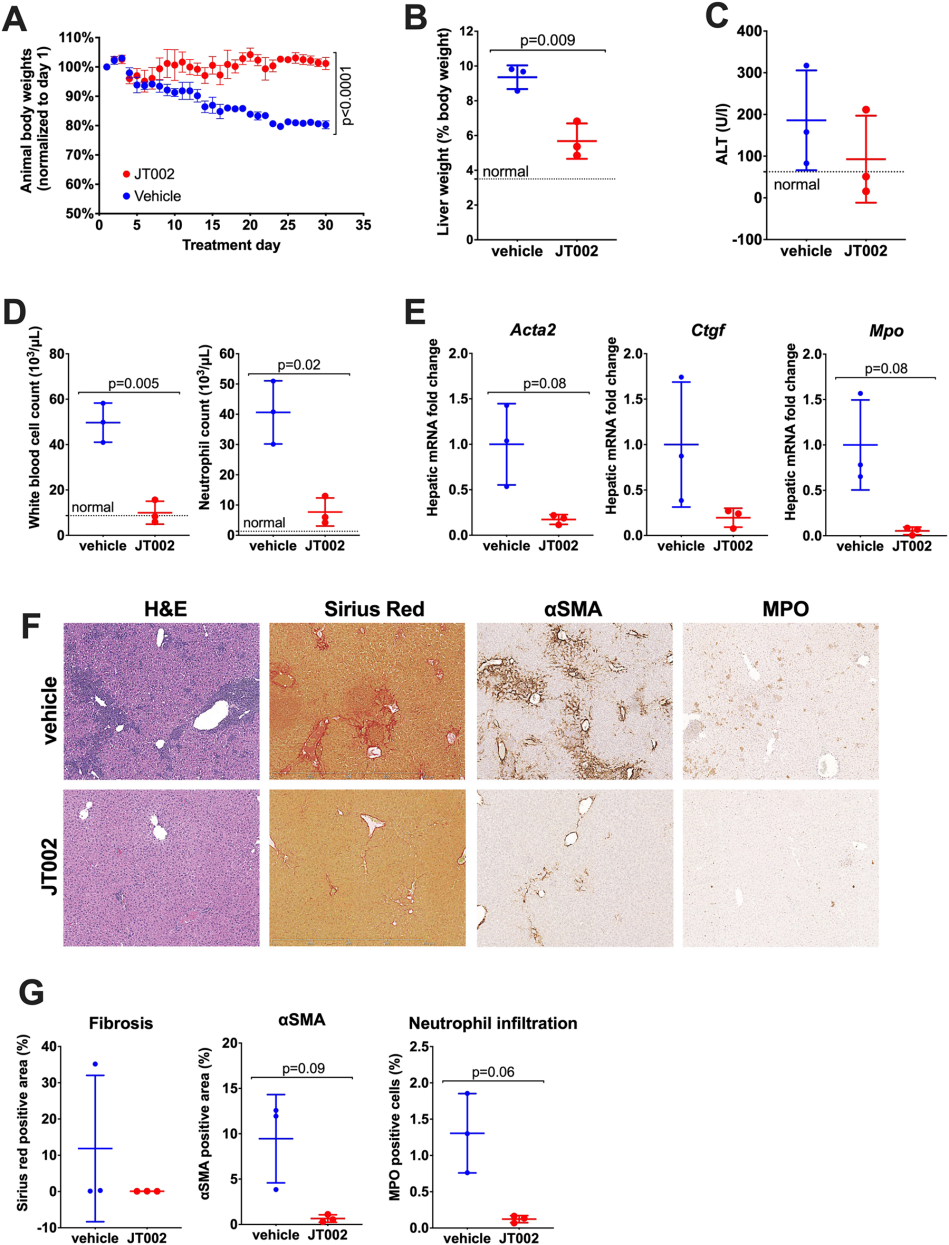
Efficacy of JT002 in the *Nlrp3*^A350V/+^ knock-in murine model of Muckle Wells syndrome. (**A**) Study timeline. (**B**) Mean $\pm$ SEM mouse body weights over the course of the study. (**C**) Normalized liver weights at study termination. (**D**) Liver alanine aminotransferase (ALT) levels at study termination. (**E**) Hepatic mRNA levels normalized to diseases control animals. (**F**) Circulating blood cell counts 1 day prior to study termination. (**G**) Quantification of histological endpoints presented in panel (**H**). (**H**) Liver histopathology at the end of the study. (**C–G**) Graphs presented show individual values with mean and standard deviation indicated. Representative normal levels of values are indicated with a dotted line.

The effect of JT002 treatment on systemic and hepatic inflammation and hepatic fibrosis was evaluated in vivo using a murine *Nlrp3* knock-in (KI) model of human Muckle Wells syndrome. In humans, mutation of alanine 352 to valine (A352V) in NLRP3 results in the constitutively active NLRP3 inflammasome which leads to excessive cytokine production and a clinical presentation characteristic of MWS^11^. The *Nlrp3*^A350V/+^ CreT KI murine model (*Nlrp3* KI model) contains a conditional A350V autosomal-dominant mutation that is analogous to the human A352V mutation^13,23,25^. In this model, mutant Nlrp3 expression occurs only in mice expressing Cre recombinase, which is inducible through the administration of tamoxifen, thus allowing for temporal control of the gain-of-function mutant *Nlrp3*. In vehicle-control mice, tamoxifen-induced activation of mutant *Nlrp*3 produces an inflammatory and fibrotic phenotype consisting of weight loss, increased liver weight, elevated liver alanine aminotransferase (ALT), systemic and hepatic neutrophilia, increased hepatic collagen deposition, and increased numbers of activated hepatic stellate cells (HSCs)^13,23,25^. In the *Nlrp3*^A350V/+^ CreT study, vehicle or 30 mg/kg JT002 (n = 4/group) was dosed orally once daily starting one day prior to the first administration of tamoxifen. One animal from each group was found dead on day 4 and day 11 from the JT002 dosed group and the vehicle group, respectively, leaving only 3 animals per group. As shown in Fig. 4, JT002 treatment prevented the body weight loss (Fig. 4A) and significantly reduced the liver weight as a percent of body weight from 9.4 to 5.7% (Fig. 4B). Serum levels of ALT in vehicle-treated mice averaged 186 U/L while ALT levels were reduced to 93 U/L in mice administered JT002 (Fig. 4C). Expression of mutant *Nlrp3* also induced systemic inflammation and neutrophilia and treatment with JT002 achieved significant normalization of circulating total white blood cell and neutrophil counts with a strong trend for reduced eosinophil counts (Fig. 4D). We analyzed hepatic expression of pro-inflammatory and profibrotic genes in vehicle and JT002 treated animals and discovered that genes linked to fibrosis such as *Acta2* (alpha-smooth muscle actin) and *Ctgf* (connective tissue growth factor) were substantially downregulated in liver tissue from JT002-treated animals (Fig. 4E). Hepatic gene expression of the neutrophil marker, myeloperoxidase (*Mpo*), and components of the inflammasome pathway, including *Pycard* (ASC), *Casp1* (caspase-1) and *Il1b* (IL-1β) were also substantially reduced in the JT002-treated mice (Fig. 4E, Supplementary Fig. 3). Histopathological assessment of liver tissue by hematoxylin and eosin (H&E) staining revealed a visible reduction in the degree of inflammation in JT002-treated mice (Fig. 4F) and hepatic immunostaining for myeloperoxidase showed that neutrophil recruitment was substantially reduced by 91% in the JT002 treated group (Fig. 4F,G). Additionally, treatment with JT002 considerably reduced hepatic collagen content as assessed by Picrosirius red staining and reduced HSC activation as shown by a 93% reduction in immunostaining for alpha-smooth muscle actin (αSMA) (Fig. 4F,G).

### JT002 attenuates airway inflammation and airway hyperresponsiveness in a mouse model of IL-17-dependent neutrophilic asthma

**Figure 5:**
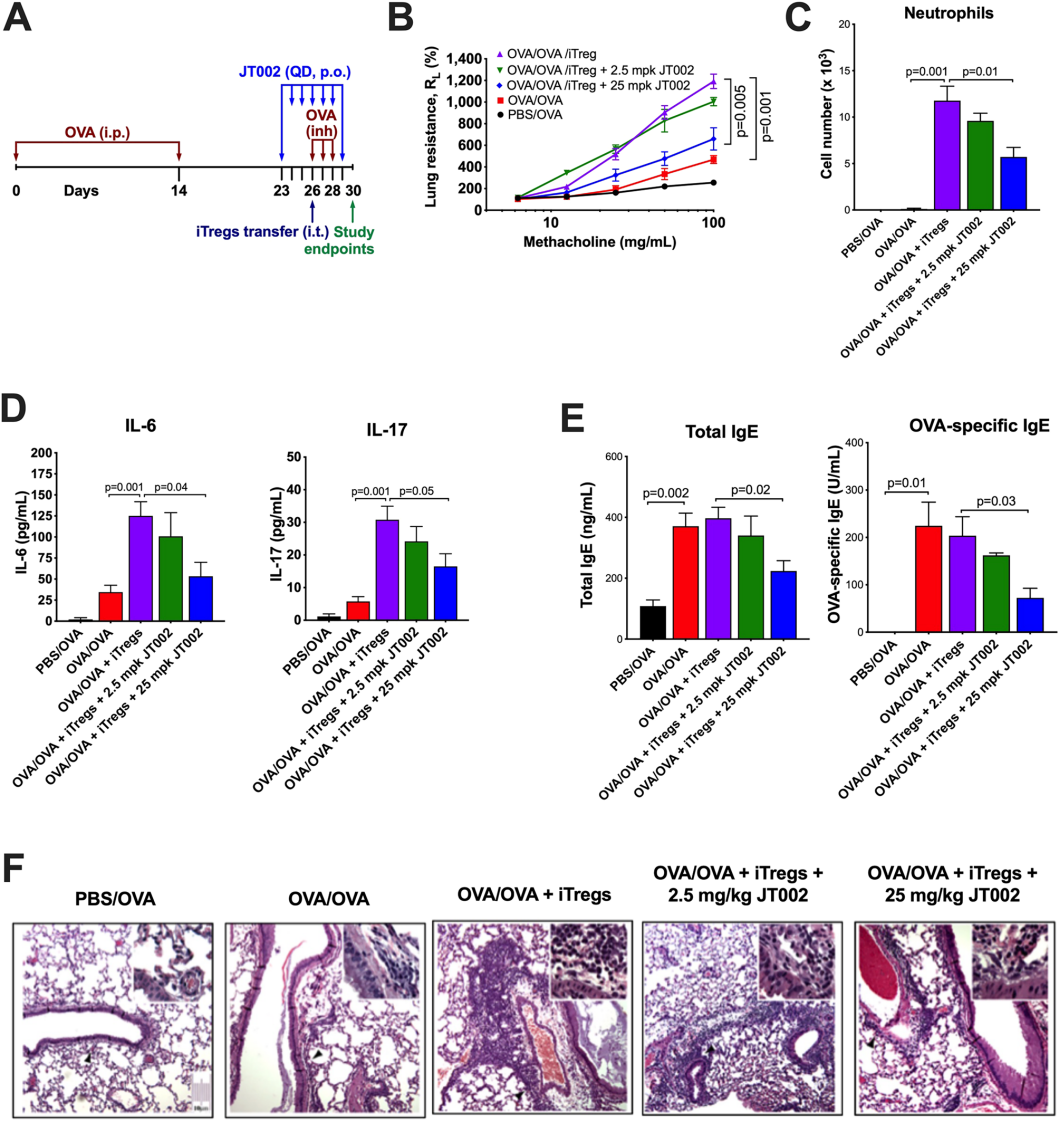
Efficacy of JT002 in the iT_reg_ induced model of neutrophilic asthma. (**A**) Study timeline schematic. (**B**) Airway hyperresponsiveness as measured by lung resistance in response to methacholine treatment in study animals. (**C**) Neutrophil cell counts in bronchoalveolar lavage. (**D**) Cytokine levels in bronchoalveolar lavage. (**E**) Serum total immunoglobulin E (IgE) and OVA-specific IgE concentrations. (**F**) Representative hematoxylin and eosin-stained lung sections. (**B–E**) Graphed data shows mean $\pm$ SEM.

In an allergen-dependent, inducible regulatory T cell (iTreg) model of IL-17-dependent neutrophilic asthma, ovalbumin (OVA) sensitized recipient CD8α^−/−^ mice are administered iTreg cells intratracheally (i.t.) on Day 26 after the first OVA challenge and subsequently challenged with OVA on Days 26–28^48^ (Fig. 5A). This model elicits T_H_17-dependent IL-17 release that promotes airway neutrophilia and enhanced airway hyperresponsiveness (AHR) to inhaled methacholine. We investigated the effect of JT002 at two doses in OVA sensitized, OVA challenged and iTreg transferred CD8α^−/−^ mice. JT002 was administered to mice once daily (QD) by oral gavage (p.o.) on Days 23–29 at 2.5 mg/kg (n = 3/group) and at 25 mg/kg (n = 7/group) before evaluation of airway hyperresponsiveness (AHR) and airway inflammation on Day 30 (Fig. 5A). Additional control groups included vehicle treated disease animals (OVA/OVA + iTregs; n = 7/group), OVA sensitized, OVA challenged control animals (OVA/OVA; n = 6/group) and phosphate buffer saline (PBS) sensitized, OVA challenged animals (PBS/OVA; n = 4/group). Compared to the OVA sensitized and OVA challenged (OVA/OVA) CD8α^−/−^ mice, the recipients of the iTreg cells showed a significant increase in AHR, airway neutrophilia and eosinophilia and a visible increase in submucosal inflammatory cell infiltration (Fig. 5B,C,F, Supplementary Fig. 4A). The increase in airway inflammation observed after instillation of iTregs correlated with increased bronchoalveolar lavage (BAL) concentrations of the pro-inflammatory cytokines IL-6 and IL-17, with no significant changes in BAL IL-10 or INFγ concentrations (Fig. 5D, Supplementary Fig. 4B). Treatment of OVA/OVA + iTreg mice with 25 mg/kg JT002 significantly decreased AHR and airway neutrophilia and visibly decreased submucosal inflammatory cell infiltration with no effect on airway eosinophilia (Fig. 5B,C,F, Supplementary Fig. 4A). In addition, JT002 dosed at 25 mg/kg significantly decreased the concentrations of BAL IL-6 and IL-17 and increased BAL INFγ concentrations, with a strong trend for increased BAL IL-10 concentrations (Fig. 5D, Supplementary Fig. 4B). There were no significant changes in AHR or airway inflammation after treatment with the low dose (2.5 mg/kg) of JT002 (Fig. 5, Supplementary Fig. 4). Sensitization and challenge with OVA led to an increase in total serum IgE as well as OVA-specific serum IgE, IgG1 and IgG2b even in the absence of iTreg instillation (Fig. 5E, Supplementary Fig. 4C). Despite the fact that there was no additional increase in OVA-specific serum Ig in sensitized and challenged mice instilled with iTregs, high dose JT002 resulted in a significant decrease in serum total IgE and OVA-specific IgE with no significant effect on OVA-specific IgG isoforms (Fig. 5E, Supplementary Fig. 4C).

### JT002 prevents airway hyperresponsiveness and airway neutrophilia in an ozone model of neutrophilic asthma

**Figure 6:**
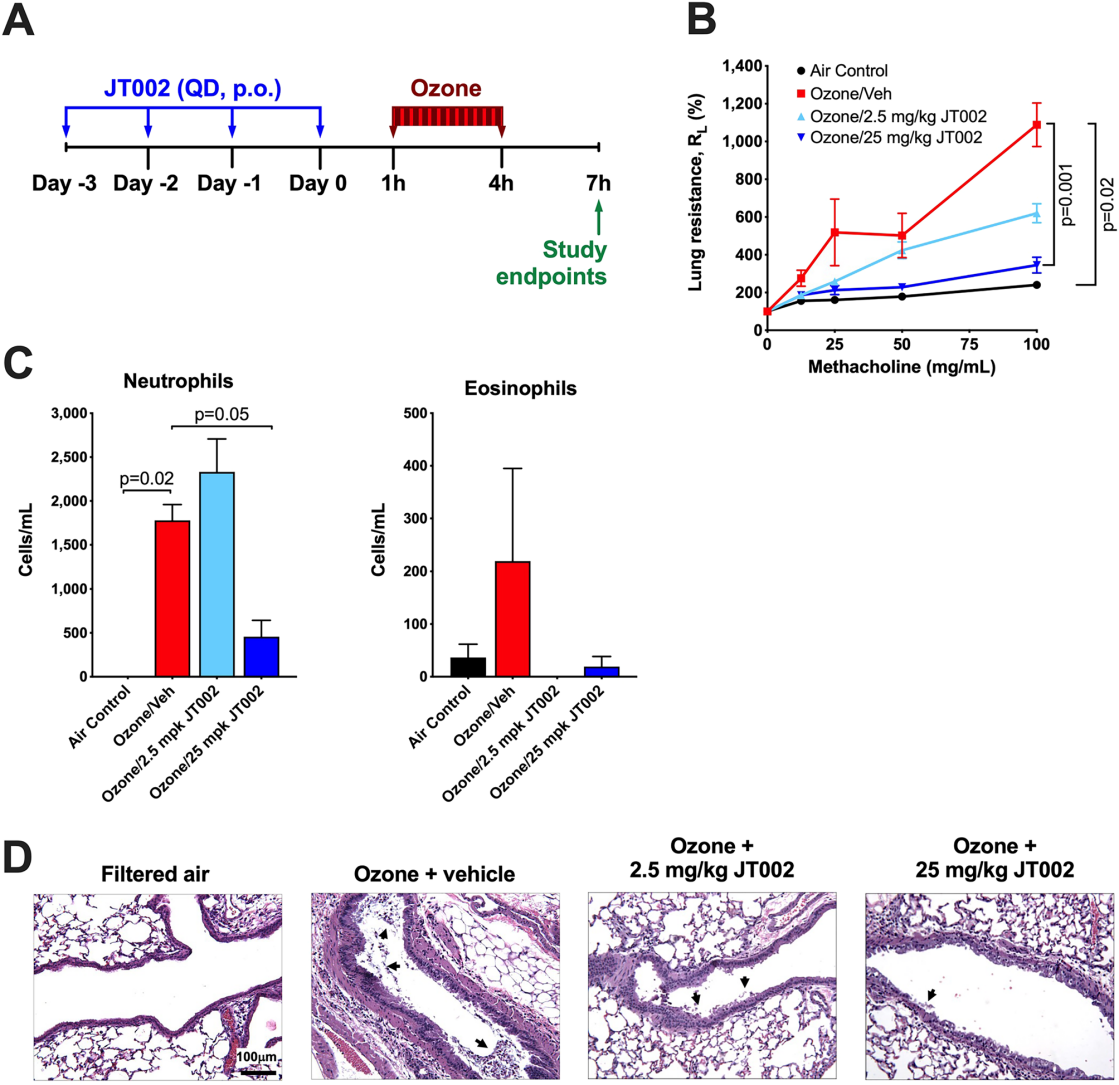
Efficacy of JT002 in the ozone induced model of neutrophilic airway inflammation. (**A**) Study timeline schematic. (**B**) Airway hyperresponsiveness as measured by lung resistance in response to methacholine treatment in study animals. (**C**) Neutrophil and eosinophil cell counts in bronchoalveolar lavage. (**D**) Representative hematoxylin and eosin-stained lung sections. (**B,C**) Graphs show mean $\pm$ SEM.

Airway epithelial cell damage caused by acute O_3_ exposure results in the release of pro-inflammatory mediators, including IL-1β, which contributes to airway neutrophilia and the development of AHR^49^. We investigated the effect of oral administration of JT002 at one of two doses in a model of ozone induced neutrophilic airway inflammation (Fig. 6A). Acute ozone exposure induced the development of AHR and significantly increased the numbers of epithelial cells and neutrophils in the BAL fluid (Fig. 6B,C, Supplementary Fig. 5). Histological analysis of lung sections showed increased epithelial cell damage in the ozone exposed mice characterized by desquamation and the appearance of epithelial cells in the airway lumen (Fig. 6D, arrows). Treatment with 25 mg/kg JT002 prevented ozone-induced AHR, significantly reduced BAL fluid neutrophil numbers (Fig. 6B,C) and visibly reduced the appearance of epithelial cells in the airway lumen, although there was no effect on the number of epithelial cells in the BAL fluid (Fig. 6D, Supplementary Fig. 5). Treatment of mice with the lower dose of 2.5 mg/kg JT002 showed a strong trend for reduction in AHR and prevention of airway epithelial desquamation, but had no effect on BAL neutrophilia (Fig. 6B–D).

## Discussion

Biologic agents targeting the IL-1β pathway, such as the recombinant IL-1 receptor antagonist, anakinra, the neutralizing anti-IL-1β antibody, canakinumab, and the soluble decoy IL-1 receptor/IL-1 receptor accessory protein chimera, rilonacept, are currently approved for the treatment of a number of inflammatory conditions including RA, CAPS and gout^50^. Canakinumab has been shown to be efficacious in the treatment of cardiovascular disease, but an associated risk of infections has also been noted, and the Food and Drug Administration (FDA) declined its approval for cardiovascular risk reduction in 2018^51^. Selectively inhibiting NLRP3 activation with a small molecule has the potential for an improved safety profile since other inflammasomes (NLRP1, NLRC4, AIM2 etc.) remain competent to mediate an immune response and produce IL-1β in the event of invading pathogens. Additional advantages include the inhibition of IL-18 production and pyroptotic cell death, as well as the convenience of oral administration. MCC950, a well-characterized, selective small molecule inhibitor of NLRP3 inflammasome activation was advanced to the clinic, but its development was terminated due to drug induced liver injury (DILI) detected at a daily dose of 1200 mg^52^. High lipophilicity and high daily dose are associated with increased risk of DILI in the clinic^53^. Moreover, MCC950 contains a furan moiety that may have contributed to its observed toxicity^54^. We discovered and herein characterize JT002, a novel small molecule inhibitor of the NLRP3 inflammasome that has lower lipophilicity than MCC950 and lacks the potentially toxicophoric furan.

In a range of primary cell-based assays, JT002 showed single digit nanomolar potency in inhibiting cytokine production and pyroptosis using a variety of stimuli. In human whole blood experiments, JT002 was threefold more potent in inhibiting IL-1β produced via the alternative pathway as compared to the canonical pathway. However, it is not entirely clear which potencies are the best predictors of therapeutic efficacy and whether efficacious doses would vary depending on the disease setting and the underlying mode of NLRP3 activation. Importantly, JT002 displayed good selectivity towards inhibiting NLRP3 inflammasome activation and did not interfere with either TLR4-mediated activation of the NF-κB pathway or other inflammasomes tested, such as NLRP1, NLRC4 or AIM2. Although the direct target of JT002 has not yet been identified, a recent publication that utilized cryo-EM demonstrated direct binding of a structurally similar compound to an allosteric pocket in the NACHT domain of NLRP3, thus explaining the inflammasome selectivity profile^55^.

We have demonstrated excellent target engagement of JT002 in a mouse pharmacodynamic model that aligned well with PK and the in vitro whole blood potency. Target engagement translated into in vivo efficacy of JT002 in multiple animal models. Daily treatment with JT002 at 30 mg/kg administered by oral gavage to mice expressing the *Nlrp3* A350V mutation resulted in the attenuation of inflammation and hepatic fibrosis compared to vehicle control mice and maintained total white blood cell counts and ALT levels within normal ranges. Low animal numbers made it difficult to reach statistically significant values, but the trends were clear across all biomarkers. We further investigated the potential of JT002 as a therapeutic agent in neutrophilic airway inflammation. JT002 showed activity in both acute and chronic models, with the results indicating that NLRP3 inflammasome activation plays a critical role in IL-17-dependent neutrophilic asthma and ozone-induced airway inflammation and supports the notion that targeting NLRP3 with JT002 is a valid approach in treating severe, steroid-resistant neutrophilic asthma.

While we have made diligent efforts to design and execute the research with rigor, some limitations of these studies still exist. For example, while we provide robust in vitro characterization showing JT002 selectively inhibits the NLRP3 inflammasome, we have not proven that the molecule binds directly to NLRP3. Identification of the direct binding target of JT002 through pull-down experiments and structural studies would promote a better understanding of how JT002 binds and inhibits the NLRP3 inflammasome and facilitate the identification of additional inhibitors with distinct physicochemical properties. A second limitation relates to the incomplete understanding of the degree of target engagement required for maximal efficacy. Oral, once daily doses of 25–30 mg/kg JT002 showed clear efficacy in preventing disease progression in a model of Muckle Wells Syndrome and in two distinct mouse models of asthma. However, a 25 mg/kg oral dose of JT002 does not completely inhibit the NLRP3 inflammasome over the entire dosing period, as shown by the lack of inhibition of ex vivo stimulated IL-1β production at 24 h post-dosing (Fig. 3D). We have not yet tested whether more prolonged target engagement, such as that achieved after twice daily dosing, would result in improved efficacy in our animal models or whether efficacious doses would vary depending on the disease setting and the underlying mode of NLRP3 activation. Understanding the pharmacodynamic-efficacy relationship in various animal models of disease will be important as NLRP3 inhibitors transition into clinical development.

In conclusion, we have discovered a selective and potent small molecule inhibitor of the NLRP3 inflammasome and demonstrated its efficacy in murine models of CAPS and neutrophilic airway inflammation. JT002 may readily serve as a useful tool compound for nonclinical research to probe the role of NLRP3 in various pathologies, as well as a potential clinical candidate for the treatment of NLRP3 driven diseases.

## Methods

### Synthesis of JT002

Synthesis of JT002 is described in the supplementary information section accompanying this publication.

### Inflammasome activation and whole blood assays

Inflammasome activation and whole blood assays were essentially carried out as described earlier^29^. Details can be found in the supplementary information section accompanying this publication. A protocol for blood draws from healthy volunteers and use of the blood in preclinical assays was approved by Western Institutional Review Board, Inc (Puyallup, WA, USA). Informed consent was obtained from each donor prior to the first blood draw.

### Animals

All animal procedures were performed in accordance with the AWA’s national guidelines and regulations for the care and use of laboratory animals and approved by the Institutional Animal Care and Use Committee (IACUC) at National Jewish Health (Denver, CO, USA), Explora Biolabs (San Diego, CA, USA), University of California at San Diego, or Seventh Wave Laboratories (Maryland Heights, MO, USA). Pathogen-free 6–8-week-old female CD8+^−/−^ mice were obtained from existing colonies at National Jewish Health and C57BL/6 female mice obtained from Jackson Labs (Bar Harbor, ME, USA) and maintained on an ovalbumin-free diet. Male 5- to 8-week-old C57BL/6 mice were obtained from Jackson Labs and maintained on normal diet. Animals were given free access to food and water and were maintained on a 12-h light/dark schedule. All animal research described is in adherence with the ARRIVE guidelines 2.0 (https://arriveguidelines.org) to ensure transparent and thorough reporting.

### Cytokine concentration, cell viability and caspase-1 activity measurements

Cell viability was determined by addition of an equal volume of CellTiter-Glo® 2.0 reagent (Promega) to the remaining cells/supernatant in each well. Intracellular Caspase-1 activity was measured using the Caspase-Glo® assay (Promega) according to the manufacturer’s instructions. The biochemical capsase-1 assay was conducted at Reaction Biology Corporation (Malvern, USA). Human IL-1β, IL-1α, and TNFα and mouse IL-1β were measured in cell culture supernatants using homogeneous time resolved fluorescence assay kits (HTRF, Cisbio, Codolet, France). Human IL-18 was measured in cell culture supernatants using an ELISA kit (R&D Systems, Minneapolis, MN, USA). Plasma IL-1β concentrations were determined by Quantikine® ELISA (R&D Systems, Minneapolis, MN).

### Western blotting

BMDMs were activated with LPS and nigericin as described above and were lysed in situ with 50 μL of 4× NuPAGE LDS sample buffer (Fisher Scientific, Hampton, NH) + 5% β-mercaptoethanol and prepared for polyacrylamide gel electrophoresis. Samples were run on NuPAGE Bis–Tris gels (Fisher Scientific, Hampton, NH) and transferred to polyvinylidene difluoride (PVDF) membranes (Fisher Scientific, Hampton, NH) using the iBlot® 2 Dry Blotting System (Fisher Scientific, Hampton, NH). Washed PVDF membranes were blocked and then probed with the following primary antibodies: procaspase-1 + p10 + p12 [EPR16883] (Abcam, Cambridge, MA), β-actin (AC-74, Sigma-Aldrich, St. Louis, MO) and NLRP3 [Cryo-2] (AdipoGen Life Sciences, San Diego, CA). Bound primary antibodies were detected using HRP-labeled secondary antibodies and membranes were imaged with an Azure c300 imager (Azure Biosystems, Dublin, CA). Bands were quantitated using ImageJ software.

### Immunofluorescence imaging

Mouse BMDMs (2.5 × 10^4^ cells/well) were plated in Collagen-IV coated 96-well plates. On the day of the assay, cells were washed with serum-free media then primed with 100 ng/mL LPS for 3 h in DMEM/F12 (1:1) containing l-glutamine. JT002 or DMSO was added to each well and incubated at 37 °C for 30 min prior to stimulation with 10 μM nigericin for 1.5 h. Cells were briefly centrifuged, rinsed with PBS, and fixed with 4% formaldehyde in PBS overnight at 4 °C. Cells were blocked and permeabilized in PBS with 10% goat serum, 1% FBS, 0.5% Triton X-100 for 30 min at 37 °C and incubated with ASC antibody (Millipore-Sigma, Cat #04-147) overnight at 4 °C. Cells were incubated with anti-mouse IgG Alexa Fluor-488 for 1 h at 37 °C. Nuclei were stained with Hoechst 33342 in PBS for 5 min and imaged using a CellInsight CX7 High-Content Screening Platform (ThermoFisher) at Phenovista Biosciences (San Diego, CA, USA).

### RNA isolation and qPCR

Total RNA was isolated from liver tissue (~ 50 mg) via syringe homogenization in TRIzol™ and cDNA synthesized using the iScript™ cDNA Synthesis kit (Biorad, Hercules, CA, USA). Real-time PCR was performed using TaqMan Fast Advanced Master Mix (ThermoFisher) and samples were amplified using QuantStudio-7 (ThermoFisher) in accordance with manufacturers’ instructions. The following TaqMan Gene Expression Assays (cat. no. 4331182, Applied Biosystems, Foster City, CA) were used: *Acta2*, Mm00725412_s1; *Ctgf*, Mm01192933_g1; *Mpo*, Mm01298424_m1; *Pycard*, Mm00445747_g1; *Casp1*, Mm00438023_m1; *Il1b* assay ID Mm00434228_m1.

### Pharmacokinetic and pharmacodynamic analyses

Groups of male C57BL/6, 7- to 8-week-old mice were dosed orally with vehicle (n = 8/group) or 25 mg/kg JT002 (n = 8/group) in a vehicle of 0.5% methyl cellulose, 0.25% Tween 80 in 50 mM NaH_2_PO_4_/Na_2_HPO_4_ at pH 8 (PK studies) and then euthanized at 4, 8- or 24-h post-dosing. At each time point, blood samples were collected into heparin vacutainer tubes by cardiac puncture and a sample of blood from each animal was reserved for determination of JT002 plasma concentrations (Seventh Wave Laboratories, Maryland Heights, USA). The remaining blood from each animal was aliquoted into triplicate wells of a 96-well polypropylene plate at 150 µL/well. Duplicate wells were treated with 100 ng/mL LPS and incubated at 37 °C for 3 h followed by treatment with 3 mM ATP and incubation for an additional hour. The remaining well received the LPS and ATP vehicles only and served as the untreated control. Following the incubation, plates were centrifuged and plasma removed to a clean plate for analysis of IL-1β concentrations by ELISA. For each time point, IL-1β production from the unstimulated control wells of all animals was averaged and the mean value set to the baseline (0% response/100% inhibition). NLRP3-dependent IL-1β production at each time point was calculated by averaging the plasma IL-1β concentrations from the LPS + ATP treated wells from the vehicle control animals and this value served as the 100% response (0% inhibition) control.

### *Nlrp3*^*A350V/+*^ CreT knock-in mouse model of MWS

*Nlrp3*^A350V/+^ CreT knock-in mice were provided by Hal M. Hoffman, University of California, San Diego, USA. The study was performed using two randomized groups of male and female *Nlrp3*^*A350V/*+^ CreT mice (n = 4/group) between 6- and 8-weeks of age at the start of the study. Vehicle (0.5% methyl cellulose, 0.25% Tween 80 in 50 mM NaH_2_PO_4_/Na_2_HPO_4_ at pH 7.8) or JT002 at 30 mg/kg was administered orally once daily for 30 days starting on Day 1. Tamoxifen (free base) (MP Biomedicals, Solon, OH, USA) was prepared in 90% sunflower seed oil and 10% ethanol and administered at 50 mg/kg intraperitoneally (IP) daily for 4 consecutive days, starting on Day 1, followed by a booster injection on Day 16. Mice were weighed every day to monitor compound activity. On Day 29, a submandibular cheek bleed was performed to obtain whole blood for total blood cell counting. Mice were euthanized on Day 30 and blood and livers were harvested and stored appropriately. Tissue specimens were fixed in 10% formalin and subsequently embedded in paraffin for histopathological assessments, or snap frozen in liquid N_2_ and stored in low temperature freezers. Complete blood counts and differentials were conducted on a HemaVet Auto Blood Analyzer (Drew Scientific, Miami Lakes, FL, USA). In vitro quantitative determination of alanine aminotransferase (ALT) in serum was performed using the Infinity™ ALT (GPT) Liquid Stable Reagent (cat. n. TR71121, Fisher Diagnostics, Middletown, VA, USA). Sirius Red staining and immunohistochemistry for hepatic MPO and αSMA were performed as described previously^23^.

### Murine model of iTreg induced neutrophilic asthma

CD4^+^CD25^−^ T cells were enriched from spleens of naïve C57BL/6 mice to > 98% purity with MACS beads (Miltenyi Biotec, Bergisch-Gladbach, Germany). Cells were washed, counted, and resuspended to a final concentration of 4 × 10^6^ cells per mL and cultured in X-Vivo 15 (Lonza, Wakersville, MD) supplemented with 5 ng/mL IL-2 and stimulated with bound anti-CD3 and 2 μg/mL soluble anti-CD28 with 5 ng/mL TGF-β for five to seven days to generate iT_reg_s. Female CD8α^−/−^ C57BL6/j mice were sensitized by intraperitoneal injection of 20 μg OVA (Sigma Aldrich, St. Louis, MO) emulsified in 2.25 mg alum hydroxide (Imject Alum, Pierce, Rockford, IL) in a total volume of 100 μL on Days 0 and 14. Sensitized and challenged mice, denoted OVA/OVA, and non-sensitized but challenged littermates (PBS/OVA) received aerosol challenges for 20 min each day on three consecutive days (Days 26–28) with 1% OVA in PBS using an ultrasonic nebulizer (Omron, Vernon Hills, IL). On Day 26, differentiated iTregs cells generated in vitro were washed and adoptively transferred (5 × 10^5^ in 50 μL of PBS) into OVA/OVA mice, denoted as OVA/OVA + iT_reg_s. OVA/OVA + iT_reg_s mice were dosed orally with JT002 at 2.5 or 25 mg/kg once daily on Days 23–29 or with vehicle (0.5% methyl cellulose, 0.25% Tween 80 in 50 mM NaH_2_PO_4_/Na_2_HPO_4_ at pH 8).

### Murine model of ozone induced neutrophilic asthma

Four groups of 4 or 5 female C57BL6/J mice (The Jackson Laboratory, Bar Harbor, ME) at 8–12 weeks of age were housed under pathogen-free conditions and maintained on an ovalbumin-free diet. Mice were dosed orally with JT002 at 2.5 or 25 mg/kg once daily for 4 days. On the last day, 1-h post-dosing of test compound, mice were exposed to O_3_ at 1.0 ppm for 3 h in stainless steel wire cages set inside 240-L laminar flow inhalation chambers. O_3_ was generated by directing compressed medical-grade oxygen through an electrical discharge O_3_ generator (Sander Ozonizer, Model 25, Erwin Sander Elektroapparatebau GmbH, Uetze-Eltze, Germany) located upstream of the exposure chamber. Exposure to HEPA-filtered air was done in a separate chamber with age- and treatment-matched control animals.

### Endpoint measurements for neutrophilic asthma models

*Determination of airway hyperresponsiveness (AHR)* AHR was assessed on Day 30 (iTreg model) or 3 h after the O_3_ exposure (ozone model) as a change in airway resistance (cm H_2_O/mL/s) after challenge with aerosolized methacholine with using a whole body plethysmograph-based system (National Jewish Medical and Research Center, Denver, CO). Methacholine aerosol was administered by nebulization in increasing concentrations (12.5–100 mg/mL) and R_L_ was continuously computed by fitting flow, volume, and pressure to an equation of motion (Labview, National Instruments, Dallas, TX). *Bronchoalveolar Lavage* Immediately after assessment of AHR, lungs were lavaged via the tracheal tube with 1 mL of Hanks’ balanced salt solution (Gibco, Grand Island, NY). Total cell numbers were counted. Differential cell counts for neutrophils and macrophages were performed in a blinded fashion by counting at least 200 cells on cytocentrifuged preparations (Model Cytospin 3, Shandon Ltd, Runcorn, UK) stained with Leukostat (Fisher Diagnostics, Fair Lawn, NJ). Cell-free BAL fluid was stored at − 80 °C until measurements of cytokine levels by ELISA (eBioscience, San Diego, CA) were performed. *Blood Collection* Immediately following AHR measurement, mice were euthanized by exsanguination and blood collected for serum. Samples were stored at − 80 °C for subsequent measurement of immunoglobulin levels by ELISA. *Lung Histology* After BAL collection, lungs were inflated through the trachea with 1 mL of 10% formalin, removed and fixed in 10% formalin by immersion. Blocks of the lung tissue were cut around the main bronchus and embedded in paraffin blocks. Tissue sections (5 μm) were affixed to glass slides, and the slides were deparaffinized and stained with H&E for qualitative analysis of inflammatory cell influx and epithelial desquamation.

### Statistical analysis

All data were graphed and analyzed using GraphPad Prism 8.0.1 software (GraphPad, La Jolla, CA, USA). Statistical analyses were performed using unpaired, two-tailed, non-parametric Mann–Whitney test (Fig. 3B, as well as, Figs. 5B–E, 6B,C, Supplementary Figs. 2, 3 to compare control groups), non-parametric, matched pairs, two-tailed Wilcoxon test (Fig. 4B) unpaired, two-tailed, parametric, Welch’s unequal variances t-test (Fig. 4C–G), unmatched, non-parametric Kruskal–Wallis test followed by Dunn’s multiple comparison test (Figs. 5B–E, 6B,C, Supplementary Figs. 2, 3) to compare treatment groups. Data were considered significant at p ≤ 0.05.

### Supplementary Information

Supplementary Information.

## Data Availability

All data generated or analyzed during this study are included in this published article and its Supplementary Information files.
