# Author Correction: JT002, a small molecule inhibitor of the NLRP3 inflammasome for the treatment of autoinflammatory disorders

**DOI:** 10.1038/s41598-023-47251-0

**Published:** 2023-11-16

**Authors:** Geza Ambrus-Aikelin, Katsuyuki Takeda, Anthony Joetham, Milos Lazic, Davide Povero, Angelina M. Santini, Rama Pranadinata, Casey D. Johnson, Matthew D. McGeough, Federico C. Beasley, Ryan Stansfield, Christopher McBride, Lynnie Trzoss, Hal M. Hoffman, Ariel E. Feldstein, Jeffrey A. Stafford, James M. Veal, Gretchen Bain, Erwin W. Gelfand

**Affiliations:** 1 Jecure Therapeutics, San Diego, CA USA; 2 Department of Pediatrics, National Jewish Health, Denver, CO USA; 3 Division of Gastroenterology and Hepatology, Mayo Clinic, 200 First Street SW, Rochester, MN USA; 4 Department of Pediatrics, University of California San Diego, La Jolla, CA USA

Correction to: *Scientific Reports* 10.1038/s41598-023-39805-z, published online 19 August 2023

The Competing interests section in the original version of this Article was incomplete.

“G.A.-A., M.L., D.P., A.S., R.P., F.C.B., R.S., C.M., L.T., J.A.S., J.M.V., and G.B. were employees of Jecure Therapeutics when this work was performed. G.A.-A., M.L., D.P., A.S., R.P., R.S., C.M., L.T., A.E.F., J.A.S., J.M.V., and G.B. were shareholders of Jecure Therapeutics when this work was performed.”

now reads:

“G.A.-A., M.L., D.P., A.S., R.P., F.C.B., R.S., C.M., L.T., J.A.S., J.M.V., and G.B. were employees of Jecure Therapeutics when this work was performed. G.A.-A., M.L., D.P., A.S., R.P., R.S., C.M., L.T., A.E.F., J.A.S., J.M.V., and G.B. were shareholders of Jecure Therapeutics when this work was performed. A.E.F is an employee and stockholder of Novo Nordisk.”

The original Article has been corrected.

