# Supplementary material for: JT002, a small molecule inhibitor of the NLRP3 inflammasome for the treatment of autoinflammatory disorders
Source: Sci Rep. 2023 Aug 19;13:13524. doi: 10.1038/s41598-023-39805-z (PMC10439952; doi:10.1038/s41598-023-39805-z)
Supplement: Supplementary file 1 — Supplementary Information. [file 41598_2023_39805_MOESM1_ESM.pdf]

JT002, a small molecule inhibitor of the NLRP3 inflammasome for the treatment of autoinflammatory disorders

Geza Ambrus-Aikelin[1], Katsuyuki Takeda[2], Anthony Joetham[2], Milos Lazic[1], Davide Povero[1], Angelina M. Santini[1], Rama Pranadinata[1], Casey D. Johnson[3], Matthew D. McGeough[3], Federico C. Beasley[1], Ryan Stansfield[1], Christopher McBride[1], Lynnie Trzoss[1], Hal M. Hoffman[3], Ariel E. Feldstein[3], Jeffrey A. Stafford[1], James M. Veal[1], Gretchen Bain[1] & Erwin W. Gelfand[2]

Supplementary Figure 1: Effect of JT002 on Cellular IL-1β Production and ASC Speck Formation

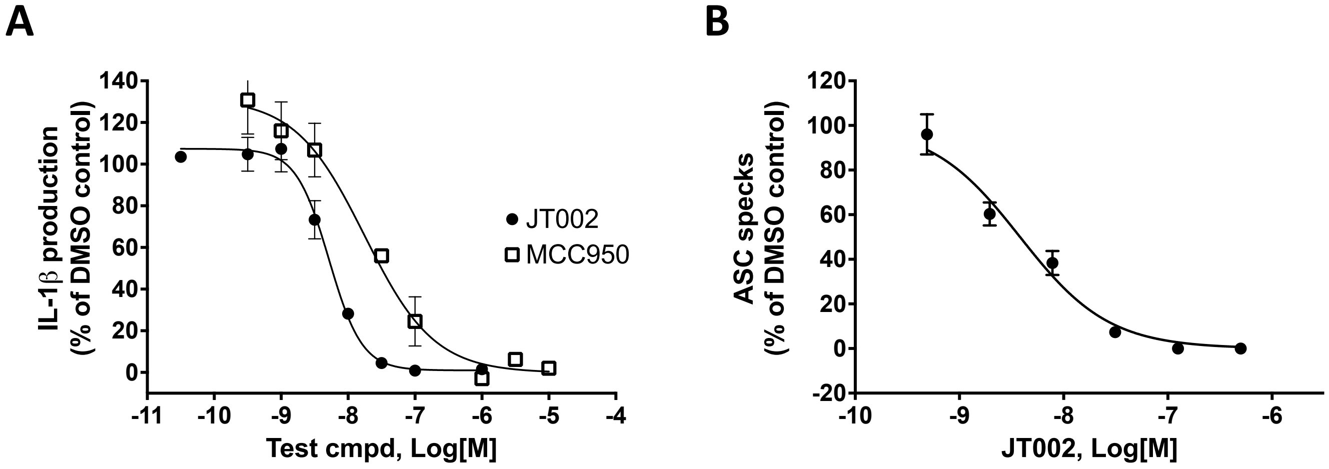

Supplementary Figure 1: (A) Inhibition of IL-1β production by JT002 vs MCC950 using human PBMCs treated with LPS + ATP. Graph shows the mean ± SEM from three independent experiments. (B) Inhibition of ASC speck formation by JT002 as measured by immunofluorescence using mouse BMDMs. Graph shows the mean ± SEM from replicate wells within a single experiment.

Supplementary Figure 2: Quantitation of Western Data from BMDMs

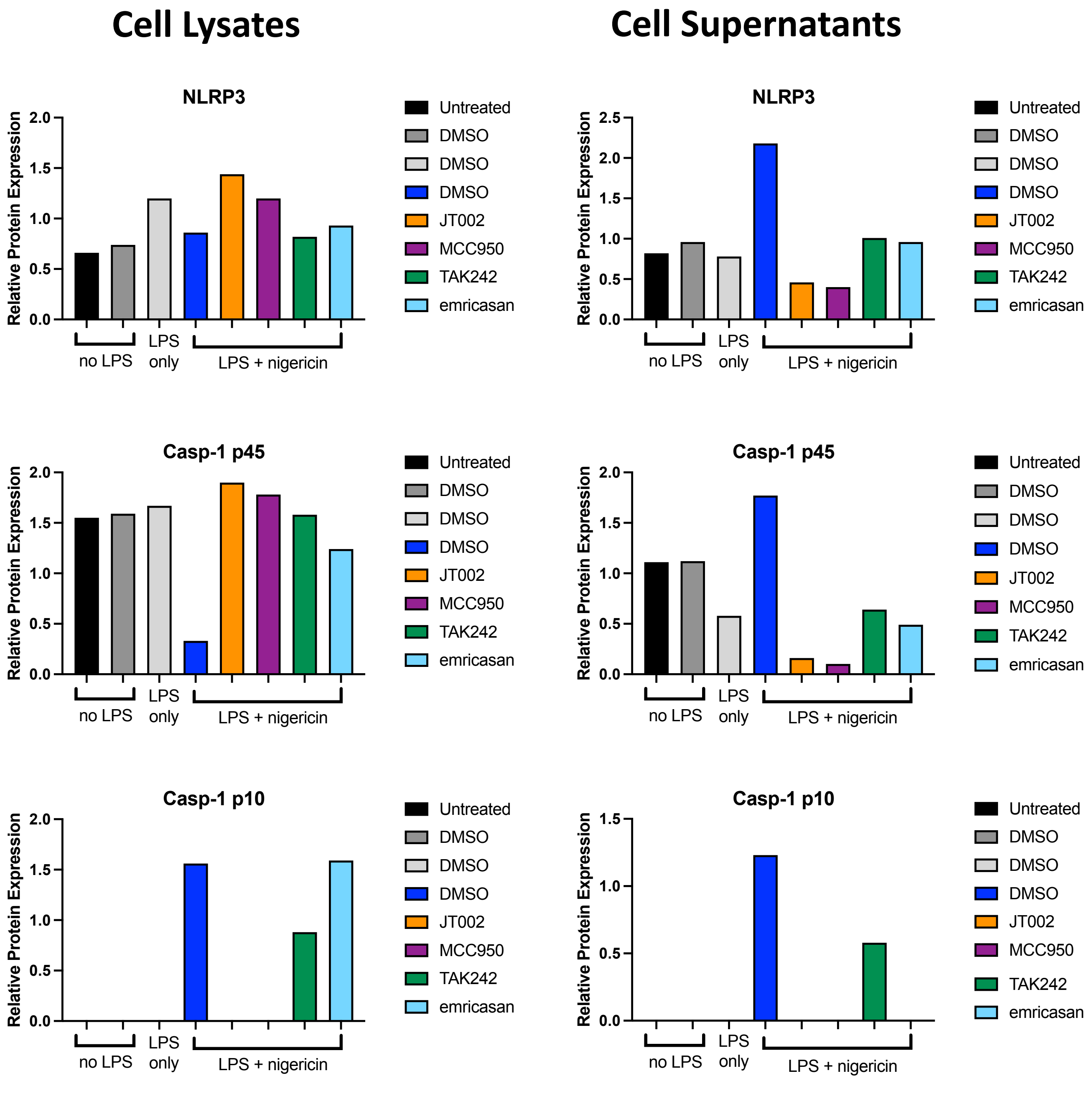

Supplementary Figure 2: Quantitation of the bands from the Western blot shown in Figure 2C. Each band was normalized to the the actin control band.

Supplementary Figure 3: Efficacy of JT002 in the A350V Knock-in Model of Muckle-Wells Syndrome

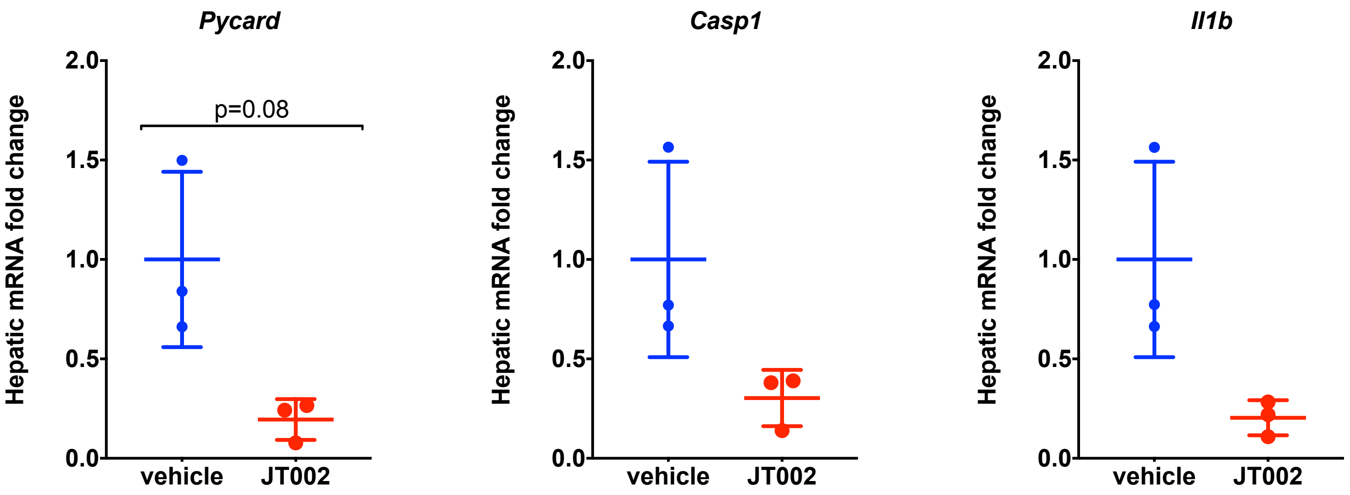

Supplementary Figure 3: Hepatic mRNA levels of inflammasome-related genes normalized to disease control. Data are graphed as Mean ± SD.

Supplementary Figure 4: Efficacy of JT002 in the iTreg Adoptive Transfer Model of Neutrophilic Airway Inflammation.

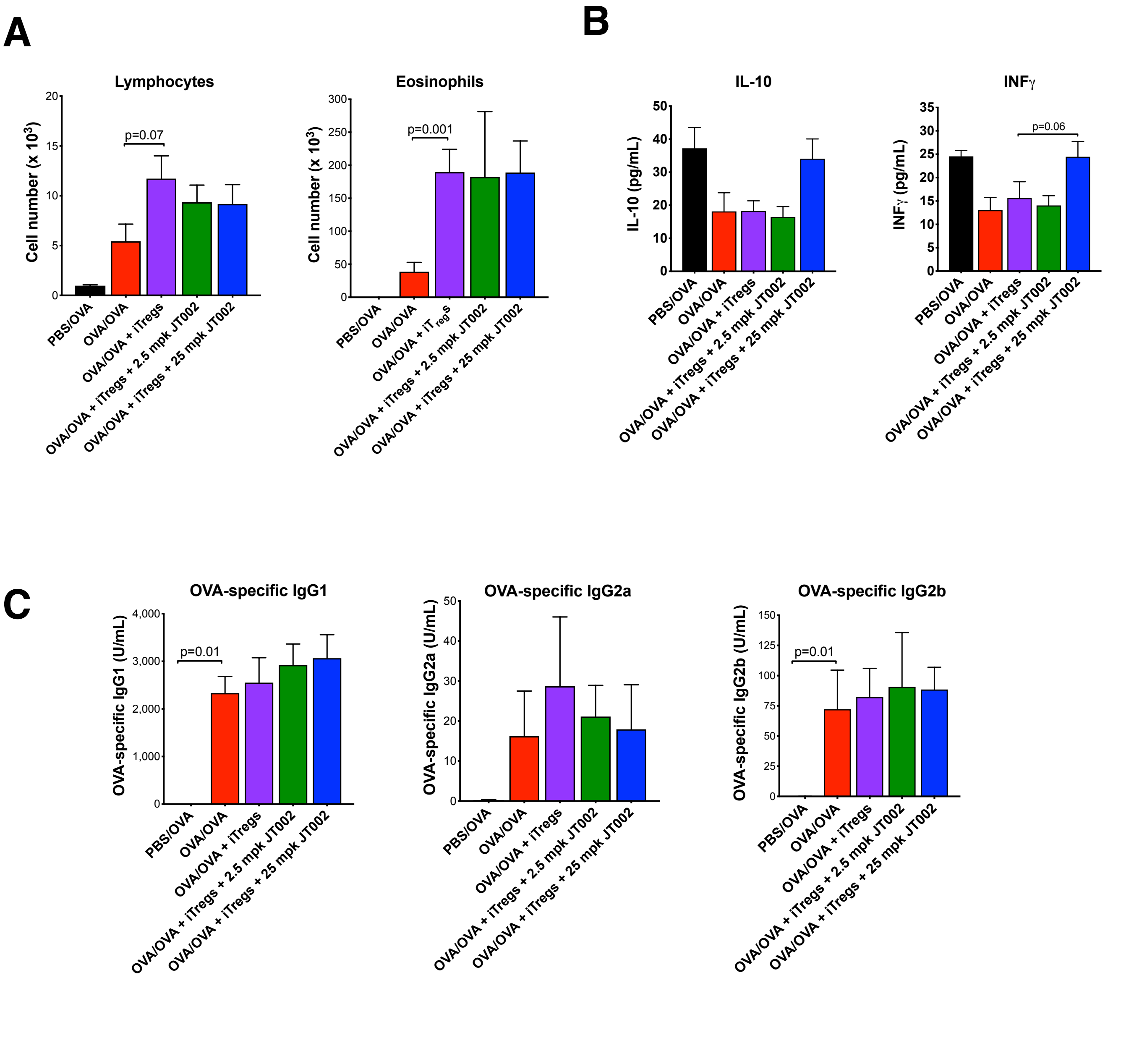

Supplementary Figure 4: Efficacy of JT002 in the iTreg adoptive transfer model of neutrophilic airway inflammation. (**A**) Leukocyte counts from bronchoalveolar lavage fluid. (**B**) Cytokine concentrations in bronchoalveolar lavage fluid. (**C**) Serum immunoglobulin concentrations. Data are graphed as mean ± SEM.

Supplementary Figure 5: Efficacy of JT002 in the Ozone Model of Neutrophilic Airway Inflammation

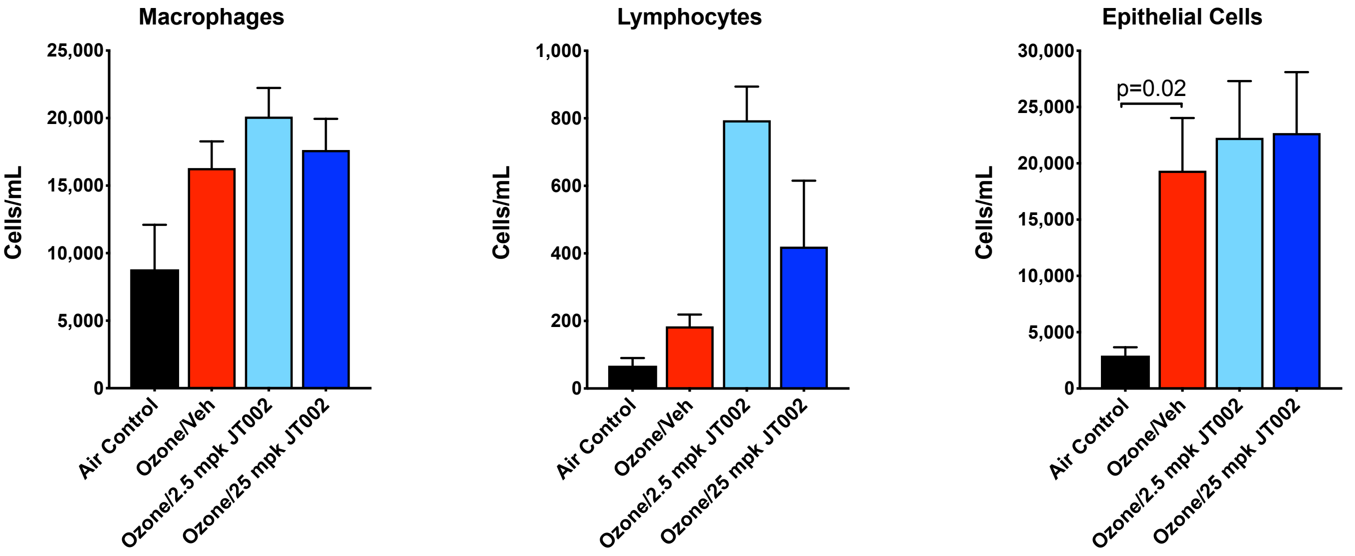

Supplementary Figure 5: Leukocyte counts from bronchoalveolar lavage fluid graphed as mean ± SEM.

Supplementary Figure 6: NLRP3 and Caspase Western Blot on Cell Lysates– Short Exposure

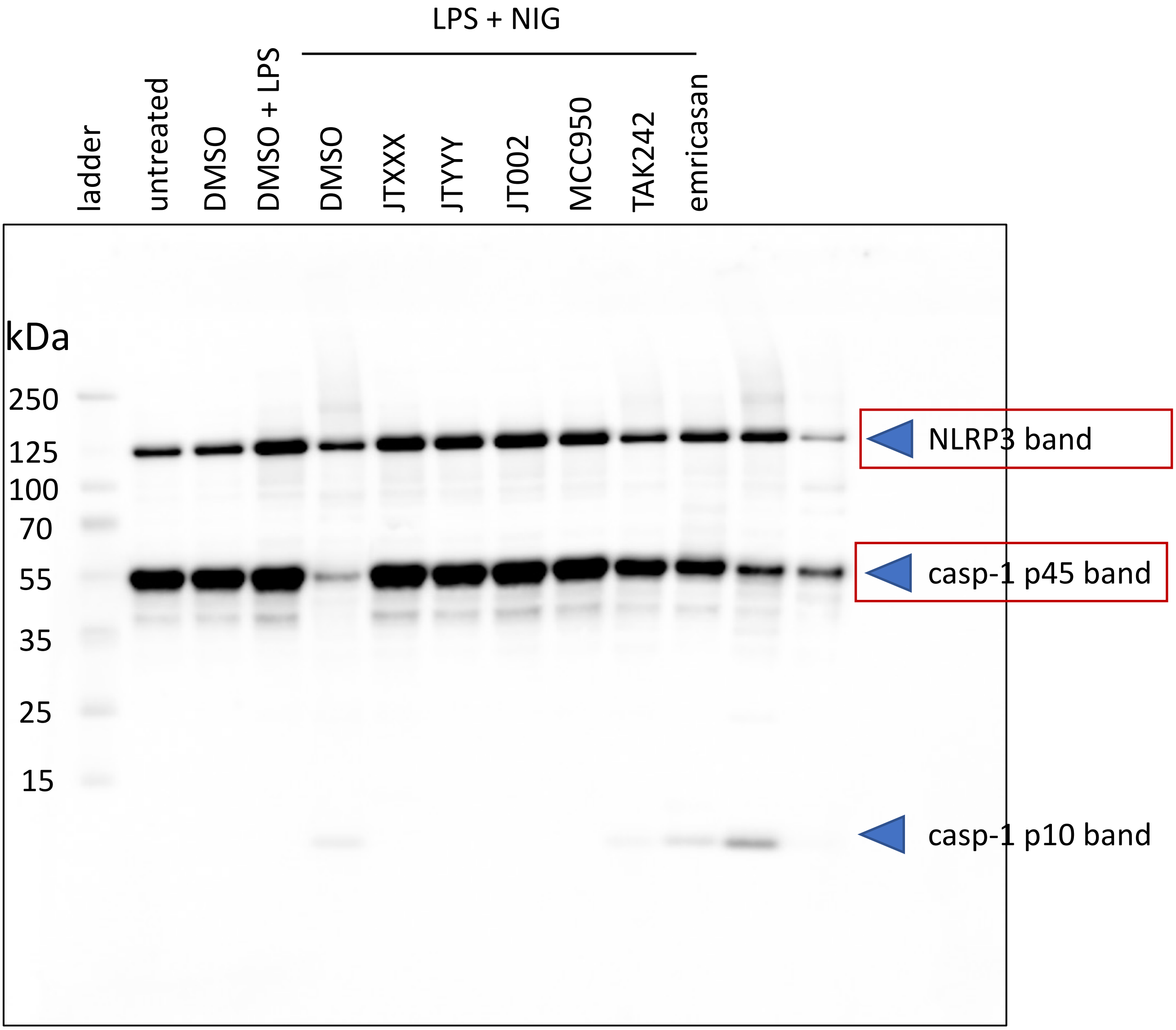

Supplementary Figure 6: Short exposure of the full blot showing the NLRP3, casp-1 p45 and casp-1 p10 bands of Figure 2C **(cell lysates blot)**. This exposure was used to generate the NLRP3 and casp-1 p45 band data in Fig 2C. Last two lanes cropped off.

Supplementary Figure 7: NLRP3 and Caspase Western Blot on Cell Lysates – Long Exposure

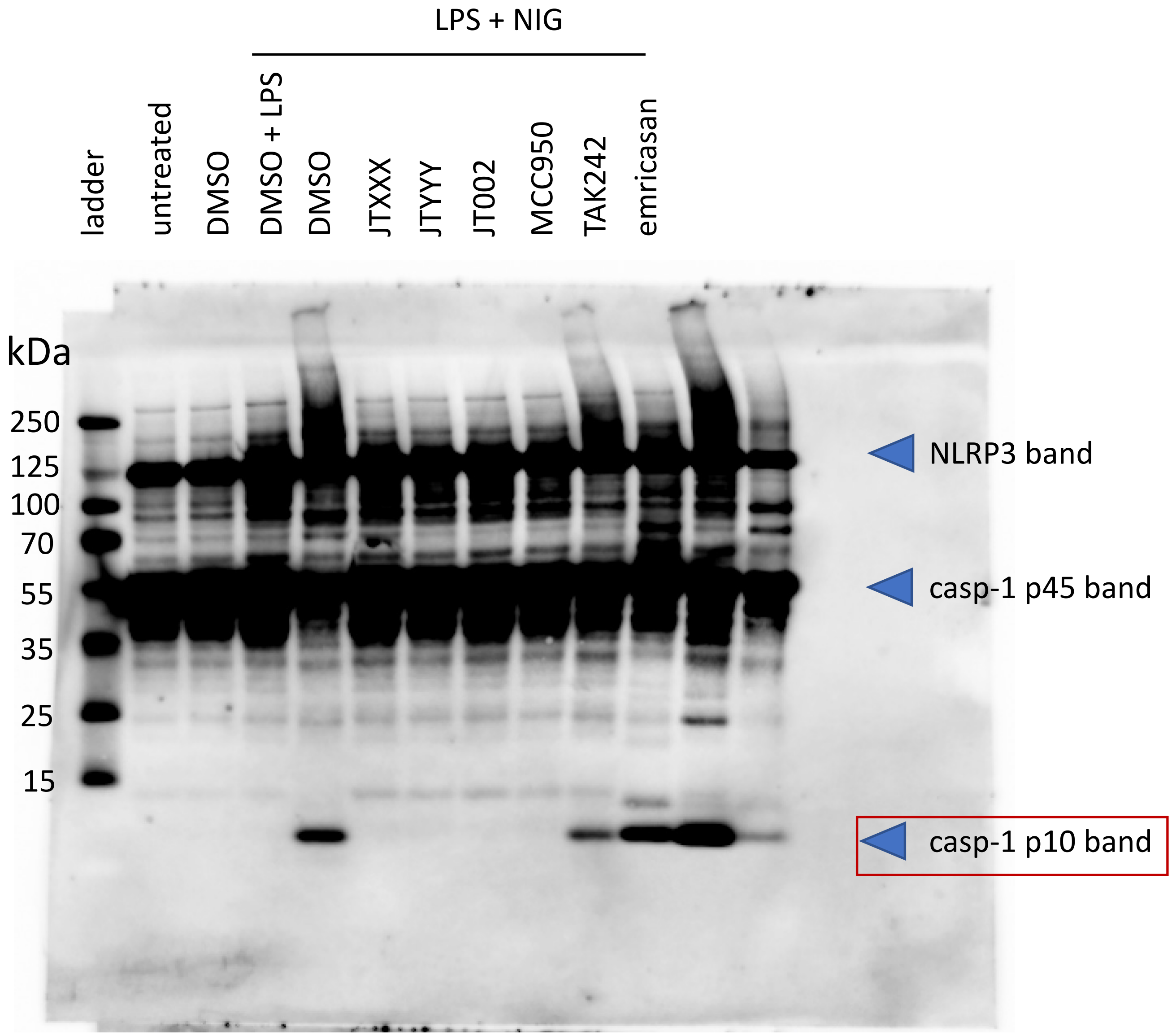

Supplementary Figure 7: Long exposure of the full blot showing the NLRP3, casp-1 p45 and casp-1 p10 bands of Figure 2C **(cell lysates blot)**. This exposure was used to generate the casp-1 p110 band data in Fig 2C. Last two lanes cropped off.

Supplementary Figure 8: NLRP3 and Caspase Western Blot on Cell Supernatants – Short Exposure

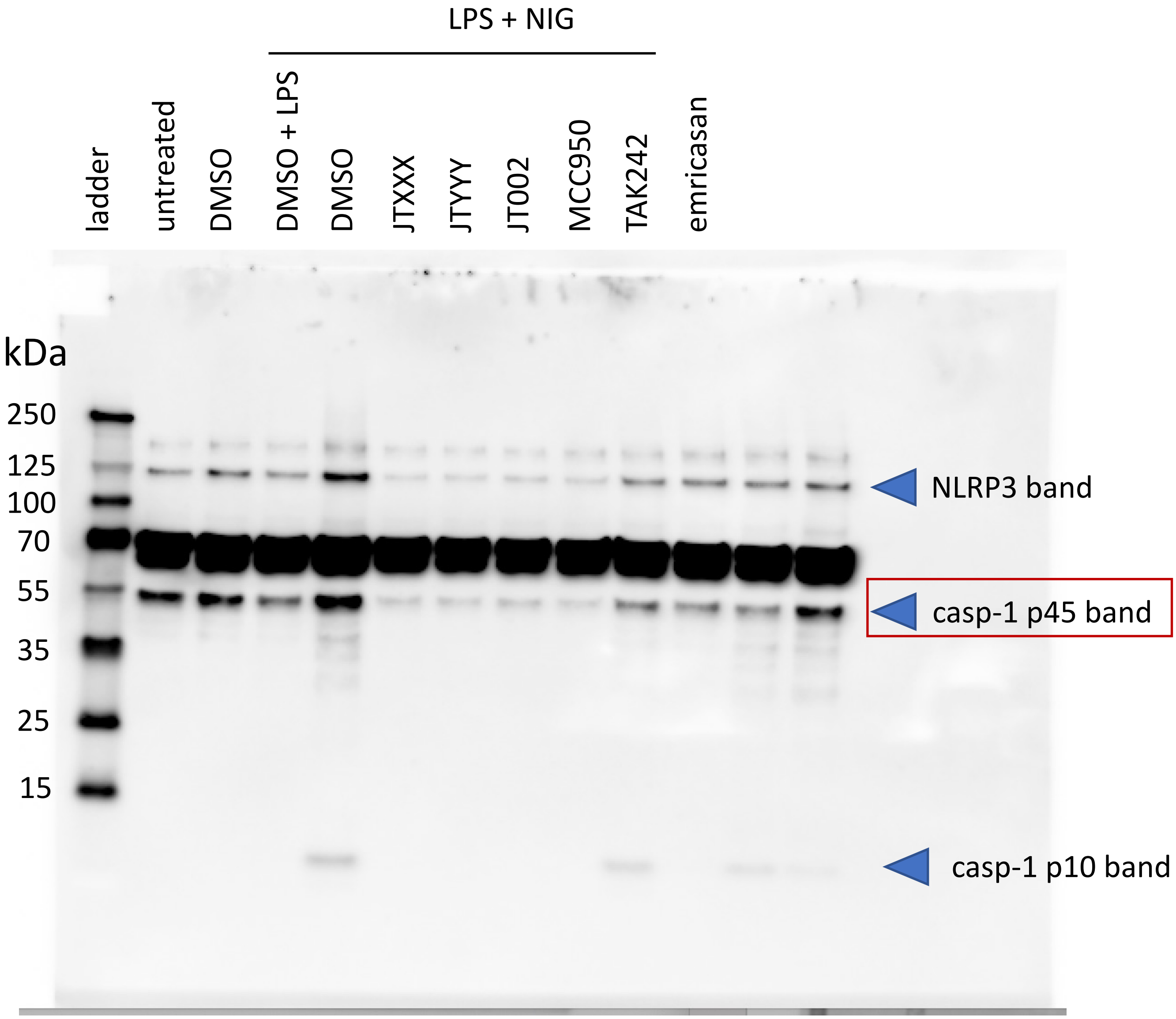

Supplementary Figure 8: Short exposure of the full blot showing the NLRP3, casp-1 p45 and casp-1 p10 bands of Figure 2C **(supernatants blots)**. This exposure was used to generate the casp-1 p45 band data in Fig 2C. Last two lanes cropped off.

Supplementary Figure 9: NLRP3 and Caspase Western Blot on Cell Supernatants – Medium Exposure

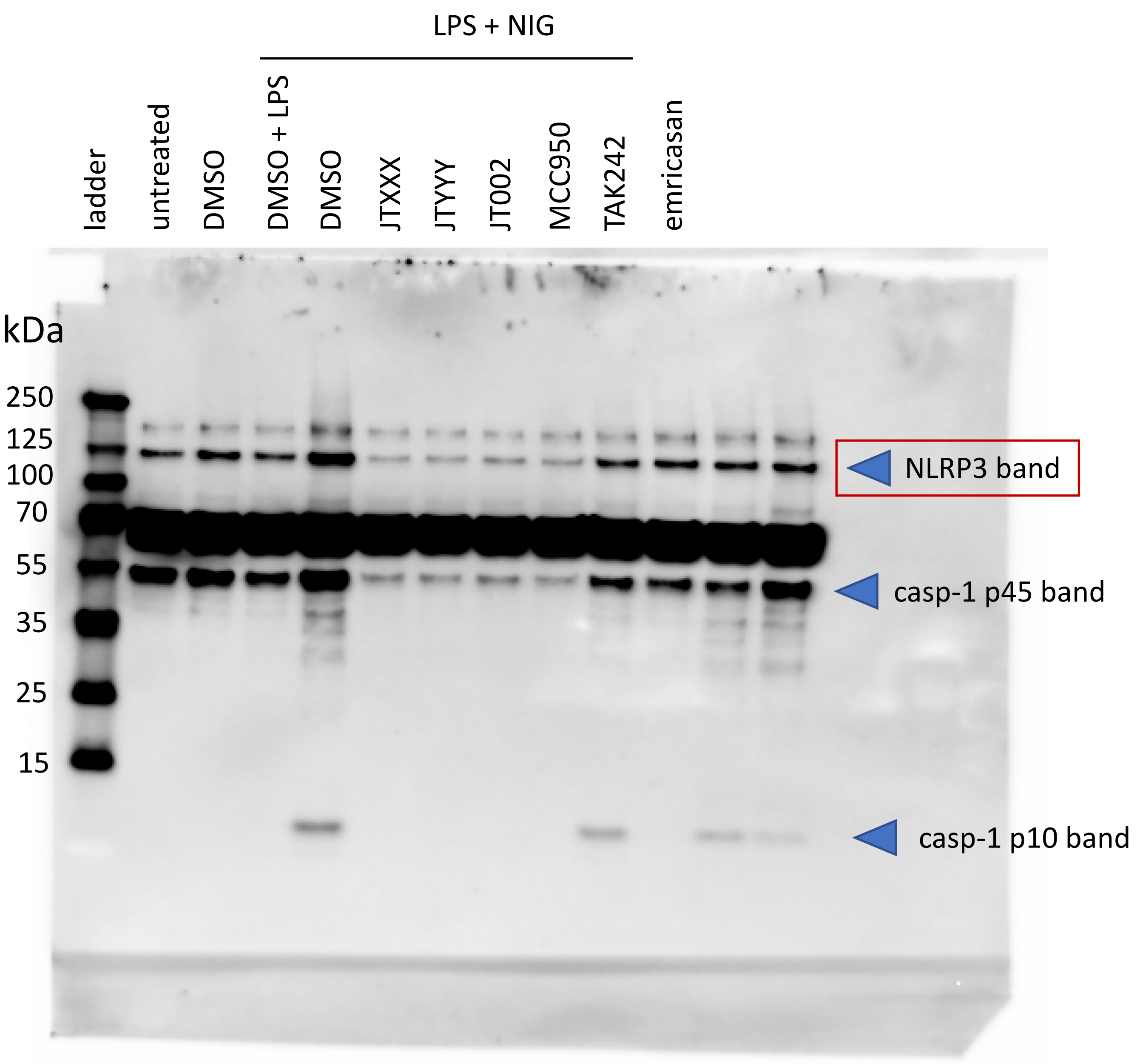

Supplementary Figure 9: Medium exposure of the full blot showing the NLRP3, casp-1 p45 and casp-1 p10 bands of Figure 2C **(supernatants blots)**. This exposure was used to generate the NLRP3 band data in Fig 2C. Last two lanes cropped off.

Supplementary Figure 10: NLRP3 and Caspase Western Blot on Cell Supernatants – Dark Exposure

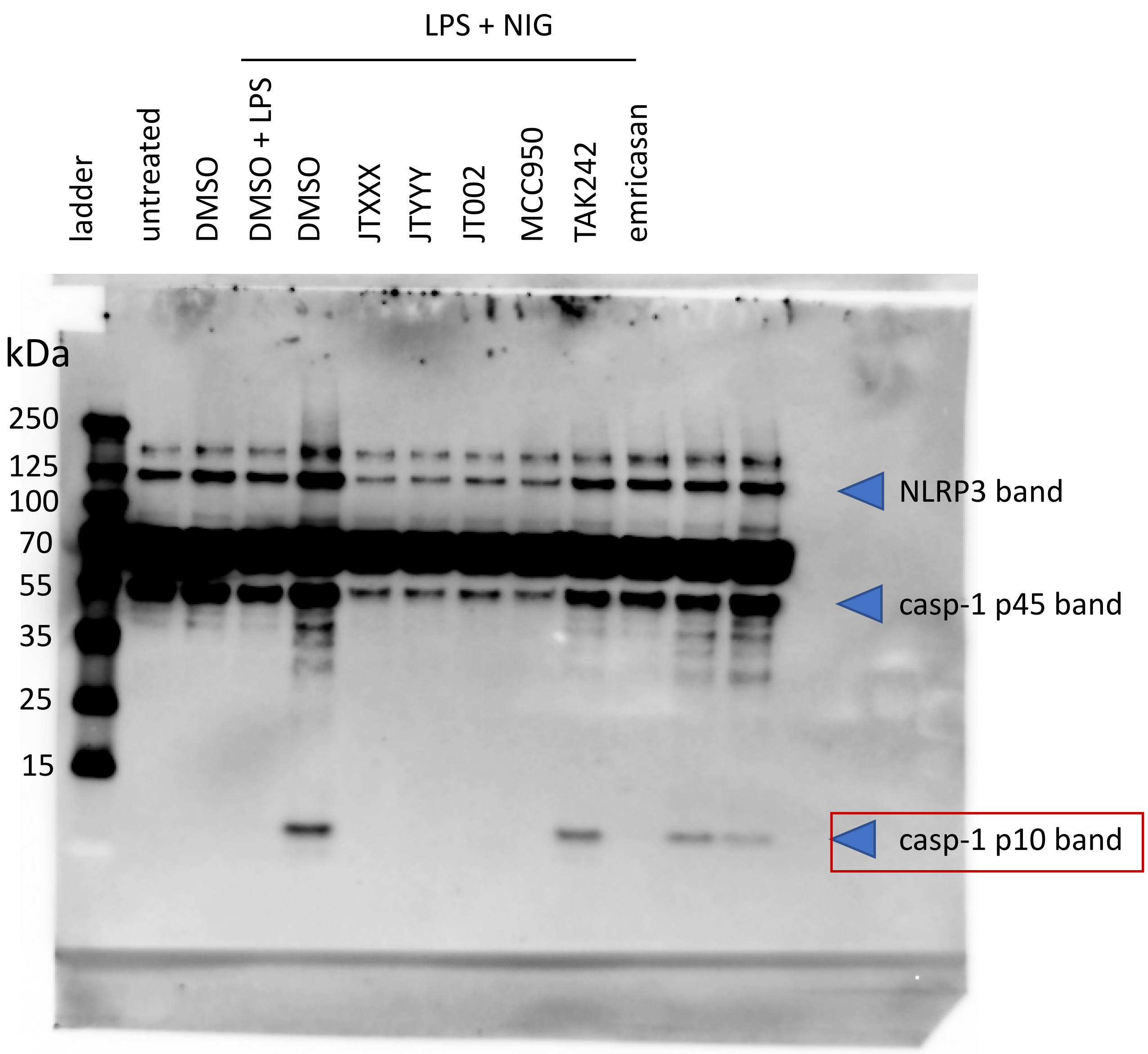

Supplementary Figure 10: Dark exposure of the full blot showing the NLRP3, casp-1 p45 and casp-1 p10 bands of Figure 2C **(supernatants blots)**. This exposure was used to generate the casp-1 p110 band data in Fig 2C. Last two lanes cropped off.

Supplementary Figure 11: Actin Western Blot on Cell Lysates – Dark and Light Exposures

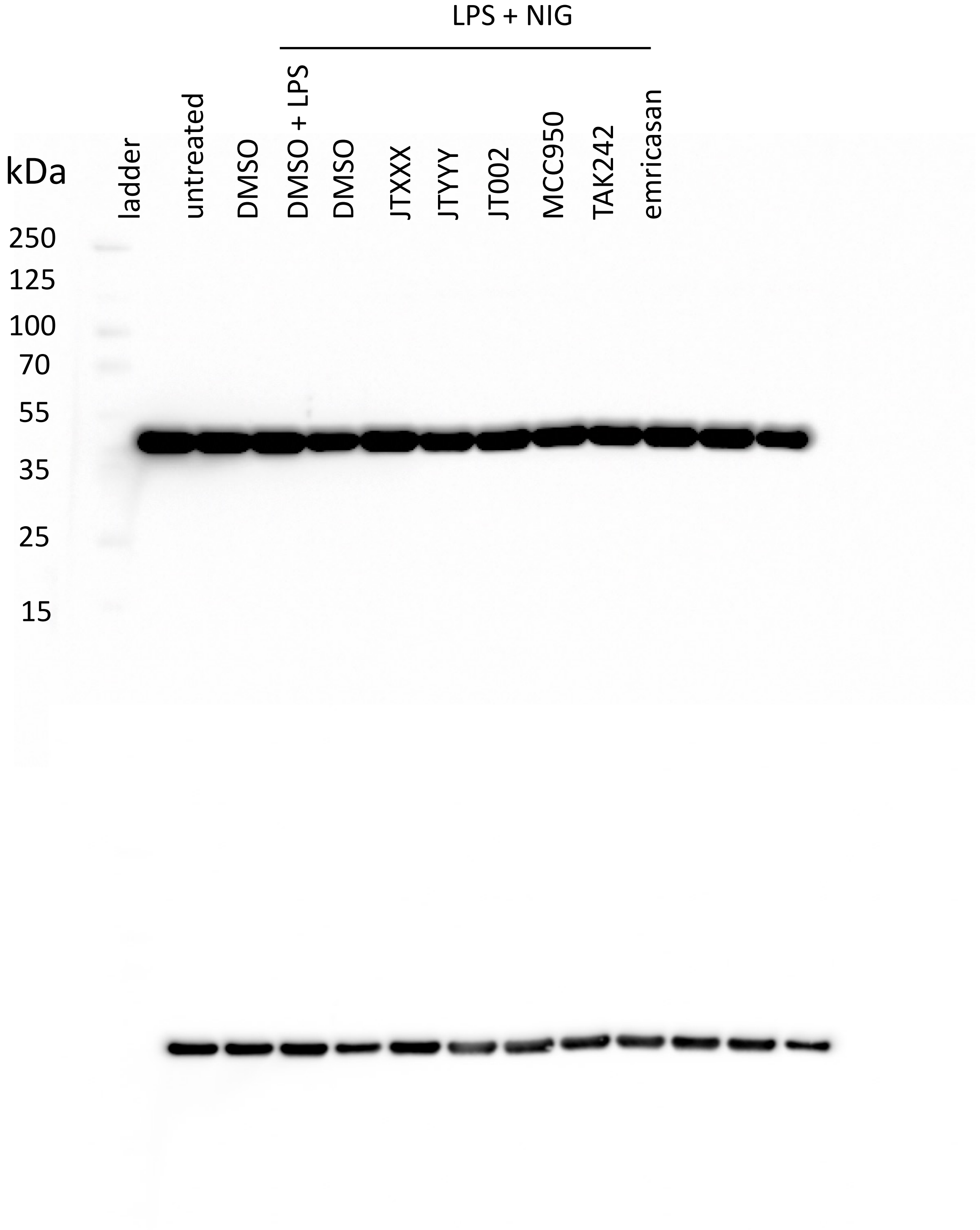

Supplementary Figure 11: Dark (upper) and lighter (lower) exposure of the full blot showing actin **(lysates blots)**. The lighter exposure was used to generate the actin band data in Fig 2C. Last two lanes cropped off.

Supplementary Methods

Synthesis of sodium (*S*)-((1,2,3,5,6,7-hexahydro-s-indacen-4-yl)carbamoyl)((6-methoxy-6,7-dihydro-5H-pyrazolo[5,1-b][1,3]oxazin-3-yl)sulfonyl)amide (JT002). (*R*)-toluene-4-sulfonic acid 3-chloro-2-hydroxy-propyl ester was prepared as described earlier[56]. Step 1: A mixture of (*R*)-toluene-4-sulfonic acid 3-chloro-2-hydroxy-propyl ester (121.8 g, 461.4 mmol), CH_3I (43 mL, 690.4 mmol) and Ag_2O (128.0 g, 551.7 mmol) in DCM (1 L) was refluxed at 45 ºC for 24 hrs. Another portion CH_3I (28.7 mL, 460.8 mmol) was added, and the reaction was refluxed for 24 hrs. Ag_2O was removed by filtration and the filtrate was concentrated, purified by silica gel column (PE/EA = 5/1) to give toluene-4-sulfonic acid 3-chloro-2-methoxy-propyl ester (77.0g, yield: 60%) as a yellow oil. ^{1}H NMR (300 MHz, $CDCl_3$): δ= 7.79 (d, *J*= 8.1 Hz, 2H), 7.35 (d, *J*= 8.1 Hz, 2H), 4.15-4.08 (m, 2H), 3.64-3.50 (m, 3H), 3.38 (s, 3H), 2.44 (s, 3H). Step 2-3: A mixture of (*R*)-toluene-4-sulfonic acid 3-chloro-2-methoxy-propyl ester (26.2 g, 94.2 mmol), 1-acetyl-1,2-dihydro-pyrazol-3-one (11.9 g, 94.2 mmol) and K_2CO_3(39.0 g, 282.6 mmol) in DMF (350 mL) was stirred at 50ºC for 16 hrs. H_2O (35 mL) was added and the reaction was stirred at 130ºC for another 3 hrs. K_2CO_3 was removed by filtration and the filtrate was concentrated, purified by silica gel column (PE/EA = 2/1) to give (*S*)-6-methoxy-6,7-dihydro-5H-pyrazolo[5,1-b][1,3]oxazine(13.8g, not pure, crude yield: 95%) as a white solid. ^{1}H NMR (400 MHz, $CDCl_3$): δ= 7.33 (d, *J*= 2.0 Hz, 1H), 5.50 (d, *J*= 2.4 Hz, 1H), 4.40-4.34 (m, 1H), 4.32-4.23 (m, 2H), 4.17 (dd, *J*= 11.6, 1.6 Hz, 1H), 3.92-3.89 (m, 1H), 3.48 (s, 3H). Step 4-5: To a solution of (*S*)-6-methoxy-6,7-dihydro-5H-pyrazolo[5,1-b][1,3]oxazine (13.8 g, 89.6 mmol) in DCM (150 mL) was added $ClSO_3H$ (13.1 mL, 197.1 mmol) dropwise at 0ºC. After being stirred at room temperature for 16 hrs, pyridine (15.8 mL, 197.1 mmol) was added dropwise at 0ºC and then PCl_5(41.0 g, 197.1 mmol) was added portion wise at 0ºC. The reaction mixture was stirred at room temperature for 1 h, poured to ice-water (200 mL) and extracted with EA(100 mL x3). The combined organic layer was washed with brine (100 mL), dried over $Na_2SO_4$and concentrated to give (*S*)-6-methoxy-6,7-dihydro-5H-pyrazolo[5,1-

b][1,3]oxazine-3-sulfonyl chloride (16.7 g, crude, yield: 74%) as a yellow solid. Step 6: To a solution of (*S*)-6-methoxy-6,7-dihydro-5H-pyrazolo[5,1-b][1,3]oxazine-3-sulfonyl chloride (16.7 g, 66.3 mmol) in THF (100 mL) was added $NH_3.H_2O$ (42 mL). After being stirred at 60 °C for 2 hrs, the reaction mixture was concentrated to about 20 mL. The residual suspension was acidified with aq.HCl (1 M) to pH = 3 and filtered. The filter cake was washed with H_2O (50 mL) and triturated with MeOH (20 mL) to give (*S*)-6-methoxy-6,7-dihydro-5H-pyrazolo[5,1-b][1,3]oxazine-3-sulfonic acid amide (10.7 g, yield: 69%) as a white solid. ^{1}H NMR (400 MHz, DMSO-d_6): δ= 7.49 (s, 1H), 7.12 (s, 2H), 4.59 (td, *J*= 11.6, 2.4 Hz, 1H), 4.32 (d, *J*= 12.0 Hz, 1H), 4.27-4.17 (m, 2H), 4.04-4.02 (m, 1H), 3.35 (s, 3H). Step 7: To a solution of 1,2,3,5,6,7-hexahydro-s-indacen-4-ylamine (15.0 g, 8.7 mmol) and TEA (13.3 mL, 95.4 mmol) in THF (30 mL), was added triphosgene (8.5 g, 28.6 mmol) in one portion at 0~5ºC and the mixture was stirred at 70 ºC under $N_2$for 1 hour. The reaction mixture was then filtered through *celite*. Then filter cake was washed with PE (30 mL). The filtrate was concentrated to dryness and dissolved in n-hexane (100 mL). The mixture was filtered through a silica gel pad. The filtrate was concentrated to dryness to give the 4-isocyanato-1,2,3,5,6,7-hexahydro-s-indacene (14.1 g, yield: 81%) as a pink oil. A suspension of (*S*)-6-methoxy-6,7-dihydro-5H-pyrazolo[5,1-b][1,3]oxazine-3-sulfonamide (1.6 g, 6.9 mmol) in MeOH (30 mL) was stirred at 80 ºC until obtaining a clear solution, then MeONa (372.6 mg, 6.9 mmol) was added and the mixture was stirred for 30 mins. The solution was concentrated to dryness and the residue was co-evaporated with MeCN (30 mL). The residual solid was suspended in MeCN (30 mL) and 4-isocyanato-1,2,3,5,6,7-hexahydro-s-indacene (1.4 g, 7.2 mmol) was added. The suspension was stirred for 16 hours at room temperature and filtered. The filter cake was triturated with PE/EA (5/1, 40 mL) to give sodium (*S*)-((1,2,3,5,6,7-hexahydro-s-indacen-4-yl)carbamoyl)((6-methoxy-6,7-dihydro-5H-pyrazolo[5,1-b][1,3]oxazin-3-yl)sulfonyl)amide (**X**, 2.4 g, yield: 80%) as a white solid. ^{1}H NMR (400 MHz, DMSO-d_6): δ = 7.42 (s, 1H), 7.36 (s, 1H), 6.76 (s, 1H), 4.50 (d,*J*= 12.0 Hz, 1H), 4.21-4.08

(m, 3H), 3.95 (s, 1H), 3.34 (overlap, 3H), 2.76 (t, *J*= 6.8 Hz, 4H), 2.67 (t, *J*= 6.8 Hz, 4H), 1.94-1.88 (m, 4H). MS: m/z 433.1 ($M+H^+$).

Inflammasome activation. PBMCs (iXCells Biotechnologies, San Diego, USA) were thawed the day before the assay and seeded in complete medium (RPMI 1640 medium with GlutaMAX, supplemented with an additional 2.5 g/L D-glucose, 10% Fetal Bovine Serum, 100 mM Sodium Pyruvate, 1% Penicillin/Streptomycin, 10 mM HEPES and 0.05 mM β-mercaptoethanol) in polystyrene V-bottom 96-well plates at $5x10^4$ cells per well and incubated overnight at 37°C with 5% CO_2. The next day, PBMC media was replaced with serum-free media prior to the assay. Differentiated mBMDMs (iXCells Biotechnologies, San Diego, USA) were plated in DMEM/F12 (1:1) with L-glutamine and 100 ng/mL M-CSF at $1x10^4$ cells/mL in 384-well tissue culture plates. The cells were incubated at 37°C, 5% CO_2 and used within 3 days. On the day of the assay, the media was replaced with DMEM/F12 (1:1) media with L-glutamine. For all assays, various concentrations of JT002, MCC950 or DMSO was added to each well and incubated for 30 minutes at 37°C, 5% CO_2. DMSO concentration was kept constant across all wells. To initiate the canonical pathway of NLRP3 inflammasome activation, cells were primed by the addition of 100 ng/mL LPS for 3-4 h, followed by the addition of either 10 μM nigericin, 5 mM ATP, 1 mg/ml cholesterol crystals, or 0.3 mg/ml monosodium urate crystals and an incubation for an additional 1.5 h, 1 h, 6 h, and 6 h, respectively. To activate the noncanonical NLRP3 pathway, mBMDMs were primed with 100 ng/mL Pam3CSK4 for 4 h followed by transfection of 2 μg/mL LPS in OptiMEM (ThermoFisher) containing 0.25% FuGENE HD and incubation overnight. To activate the NLRP1, NLRC4 and AIM2 inflammasomes mBMDMs were plated as above in 384-well plates and treated with JT002 or DMSO followed by incubation for 30 minutes prior to priming and stimulation. Cells were primed with 100 ng/mL of LPS for 3 h. For AIM2 activation, cells were stimulated for 3 h with 2 μg/mL poly(dA:dT), prepared in OptiMEM and combined with Lipofectamine 2000 (ThermoFisher). For NLRC4 inflammasome activation, cells were incubated for 3 h with 0.5 μg/mL

flagellin, prepared in OptiMEM and combined with Lipofectamine 2000. For NLRP1 activation, mBMDMs from *Nlrp3*$^{-/-}$ mice (kindly provided by Prof. Hal Hoffman, UCSD, San Diego, CA, USA) were generated (iXCells Biotechnologies, San Diego, USA). After priming *Nlrp3*$^{-/-}$ mBMDMs with 100 ng/mL of LPS for 3-5 h, cells were exposed to 100 ng/mL L18-MDP and incubated overnight. At the end of the incubation period of the assays above, plates were briefly centrifuged and half of the cell culture supernatant removed and either analysed immediately or stored at -80°C for subsequent cytokine analysis.

Whole blood assays. Fresh blood was collected into heparin vacutainer tubes, pooled and used within 1 hour of draw. Test compounds (2 µL of 100x stock in 50% DMSO/50% saline) were added to 96-well polypropylene plates followed by the addition of 198 µL of blood. For canonical stimulation of NLRP3 inflammasome, LPS (10 µL of a 2.1 µg/mL stock in saline) was added to each of the stimulated wells for a final concentration of 100 ng/mL and the plate incubated at 37°C, 5% CO_2 for 3 hours. Thereafter, ATP (10 µL of a 22x stock in saline) was added to each of the stimulated wells for final concentrations of 1 mM (human blood assay) or 3 mM (mouse blood assay) and the plate incubated at 37°C, 5% CO_2 for 1 hour. To activate the alternative NLRP3 inflammasome pathway (functional in human monocytes, but not in mice), only LPS (10 µL of a 2.1 µg/mL stock in saline) was added to each of the stimulated wells for a final concentration of 100 ng/mL and the plate was incubated at 37°C, 5% CO_2 for 6 hours. Plasma was prepared by centrifugation of the plate at 1,500 x g for 12 min.
